# The Potential Energy Landscape Formalism for Quantum Liquids in the Continuous Ring-Polymer Limit

**DOI:** 10.1021/acs.jctc.6c01077

**Published:** 2026-09-17

**Authors:** Yang Zhou, Gustavo E. Lopez, Nicolas Giovambattista

**Affiliations:** † Ph.D. Program in Physics, 2037 The Graduate Center of the City University of New York, New York, New York 10016, United States; ‡ Department of Physics, Brooklyn College of the City University of New York, Brooklyn, New York 11210, United States; § Ph.D. Program in Chemistry, The Graduate Center of the City University of New York, New York, New York 10016, United States; ∥ Department of Chemistry, Lehman College of the City University of New York, Bronx, New York 10468, United States

## Abstract

The potential energy landscape (PEL) formalism is a powerful tool within statistical mechanics to study the thermodynamic behavior of classical and quantum liquids as well as non-annealing glasses. In the quantum case, this theoretical framework is based on the path-integral formulation of statistical mechanics where the liquid/non-annealing glass is mapped onto a classical system of ring-polymers. In previous studies, and motivated by path-integral (PI) computer simulations, the PEL formalism was developed assuming that the ring-polymers were discrete, i.e., each ring-polymer was composed of a finite number of beads, *n*_*b*_. In this work, we revisit the PEL formalism for the case where the ring-polymers of the system are continuous, i.e., *n*_*b*_ → ∞ (as required in the PI formulation of statistical mechanics). We find that in this limit, the PEL of the quantum liquid is unique and well-defined. The main PEL properties of the system, including the average inherent structure energy *E*_*IS*_, configurational entropy *S*_*IS*_, and vibrational Helmholtz free energy *F*_*vib*_, are also well-defined (i.e., they converge). Notably, in the harmonic approximation of the PEL, *F*_*vib*_ can be expressed analytically; it is identical to the free energy of a multidimensional quantum harmonic oscillator with the corresponding frequencies given by the normal mode frequencies of the classical liquid counterpart (accessible from classical computer simulations). When applied to non-annealing glasses and crystals, this new version of the PEL formalism reduces to the so-called *quasi-harmonic approximation*, commonly used in the study of solids. Overall, the PEL formalism for *n*_*b*_ → ∞ provides an alternative approach to its previous version (finite *n*_*b*_); it is also more elegant and intuitive, and simpler to apply. For a Gaussian and harmonic PEL, this formalism (*n*_*b*_ → ∞) allows one to obtain the Helmholtz free energy of the quantum liquid using classical computer simulations, avoiding the more expensive PI computer simulations.

## Graphical abstract

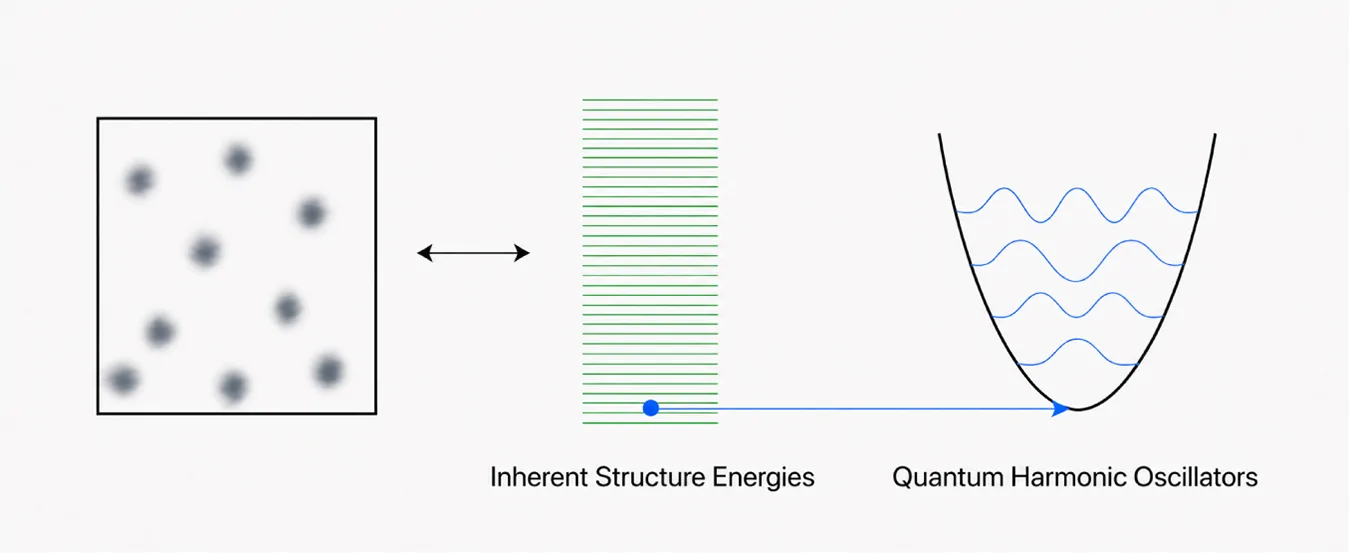

## 1 Introduction

The PEL formalism is a theoretical framework within statistical mechanics originally developed to describe *classical* liquids and glasses.^1–7^ This theoretical framework provides (A) an intuitive, qualitative description of liquids, crystals, and glasses and, (B) under simple approximations (e.g., the Gaussian approximation of the PEL), it also provides a formal, analytical expression of the system’s Helmholtz free energy *F*(*N*, *V*, *T*) which, combined with computer simulations, allows one to obtain all the thermodynamic properties of the system of interest.^4,8–10^ For a classical system composed of *N* atoms, the classical potential energy landscape (briefly, CL-PEL) is the surface in (3*N* + 1)-dimensional space defined by the potential energy of the system, *U*(*r⃗*_1_, *r⃗*_2_, ...*r⃗*_*N*_), as a function of the atomic coordinates {*r⃗*_1_, *r⃗*_2_, ...*r⃗*_*N*_}. The system (e.g., a liquid, glass, or crystal) is then represented by a point that travels with time on the CL-PEL; the representative point of the system is defined by the atomic coordinates. In the liquid state, the atoms are able to diffuse and hence, the representative point of the system moves over time defining a trajectory on the CL-PEL. Accordingly, the liquid explores different basins of the CL-PEL, each basin being characterized by a single local potential energy minimum, or inherent structure (IS). Instead, in the case of non-annealing glasses and crystals, the system is limited to move within a single basin of the CL-PEL since the atoms/molecules are limited to oscillate about fixed positions in real space.^2,3^

In the last few years, the PEL formalism has been extended to the study of systems (liquids, crystals, glasses) that obey quantum mechanics (with classical Maxwell-Boltzmann statistics, neglecting identical-particle exchange effects).^11–15^ This implies that points (A) and (B) also apply to quantum systems. Accordingly, in combination with path-integral computer simulations, the PEL formalism provides the system’s Helmholtz free energy *F*(*N*, *V*, *T*) and its thermodynamic properties.^15–18^ In the quantum case, one uses the path-integral formulation of quantum statistical mechanics (see below) where the system of interest is mapped onto a (peculiar) *classical* ring-polymer system; briefly, each atom of the quantum liquid/crystal/glass is substituted by a classical ring-polymer composed of *n*_*b*_ beads. The PEL of the quantum system is then defined as the potential energy landscape of the associated ring-polymer system (briefly, RP-PEL). Hence, as for the classical case, a quantum liquid/crystal/glass is visualized as a point that moves, over time, on the RP-PEL.

From a practical point of view, the PEL formalism for classical liquids, as well as its extension to quantum liquids, is not meant to replace computer simulations but to complement them. It provides analytical expressions for thermodynamic properties that depend on a handful of PEL variables. These PEL variables are evaluated by fitting specific properties calculated in computer simulations to the corresponding expressions predicted by the PEL formalism.^4,12,15^ Briefly, the PEL formalism provides a theoretical framework and analytical expressions that allow one to rationalize the thermodynamic behavior and properties of a given system (liquid, glass, or crystal) obtained from computer simulations or experiments. This is particularly relevant for the study of realistic classical and quantum atomistic/molecular liquids at low temperatures and close to their glass transition temperature. For example, the PEL formalism has been particularly useful in the case of water at low temperatures where classical molecular dynamics (MD) and path-integral (PI) computer simulations can be computationally very expensive or inaccessible. By combining computer simulations with the PEL formalism, it has been possible to locate water’s liquid–liquid critical point using different water models, both in the presence (PI simulations)^16,17^ or absence (classical MD simulations) of nuclear quantum effects (NQE).^8–10,19^ In such studies, one calculates the PEL properties at relatively high temperatures, where MD/PI simulations are possible, and then extrapolates the thermodynamic expressions, such as the equation of state, to very low temperatures, where direct computer simulations are computationally inaccessible. Similarly, the PEL formalism for quantum liquids has been recently applied to describe isotope effects in equilibrium and supercooled water, including water’s free energy.^18,20^ Numerous PEL studies have been performed in the past to study glass-formers such as silica and water using classical models based on empirical^9,10,21^ and, more recently, machine-learning potentials.^22^

In its present form, the PEL formalism for quantum liquids requires setting in advance the number of beads per ring-polymer, *n*_*b*_. This is not a practical limitation of the PEL approach since, indeed, the value of *n*_*b*_ needs to be set when performing path-integral computer simulations. However, from the perspective of the PEL formalism, this is not ideal. For example, at present, (i) it is unclear how PEL studies of a given system based on different values of *n*_*b*_ may compare with one another – the thermodynamic predictions are, of course, independent of *n*_*b*_ but the PEL properties may not. In addition, (ii) it is not known what properties of the PEL converge as *n*_*b*_ → ∞ and, fundamentally, (iii) whether the PEL for a quantum liquid exists at all in this limit (the path-integral formalism of quantum statistical mechanics, where the quantum liquid is mapped onto a ring-polymer system, is exact in the limit *n*_*b*_ → ∞; in this limit, the corresponding PEL would be a surface in (3*Nn*_*b*_ + 1) → ∞ dimensional space). In this work, we revisit the PEL formalism for quantum liquids and address points (i)–(iii) above. We show that for moderate values of *n*_*b*_, accessible in PI simulations, most properties of the PEL already converge. Accordingly, these PEL properties and the PEL itself are well-defined in the *n*_*b*_ → ∞ limit. Notably, for quantum liquids with a harmonic PEL, the analytical expressions derived within the PEL formalism become considerably simpler in the continuous ring-polymer limit, and all thermodynamic properties can be obtained directly from classical MD simulations (as opposed to PI computer simulations), reducing the computational cost. The predictions of the PEL formalism for *n*_*b*_ → ∞ are validated using PI computer simulations of two (quantum) Lennard-Jones binary mixtures (LJBMs) with different levels of quantumness (quantified using two different values of the Planck constant *h*; Section 3).

The structure of this work is as follows. In Section 2, we discuss the PEL formalism for quantum liquids. The ideas and equations presented in Section 2 for the case *n*_*b*_ → ∞ are validated with PI computer simulations of two (quantum) LJBMs with Planck constants *h* = *h*_1_ and *h* = *h*_4_. Computer simulation details and results are given in Sections 3 and 4, respectively. A summary and discussion are included in Section 5.

## 2 The PEL Formalism for Quantum Liquids

In Section 2.1, we review briefly the PEL formalism for quantum liquids as implemented in previous studies, where the ring-polymers are discrete, composed of a finite number of beads *n*_*b*_. In Section 2.2, we extend the PEL formalism to the case where *n*_*b*_ → ∞ and hence, the ring-polymers become continuous, analogous to elastic bands.

### 2.1 Overview of the PEL Formalism for Quantum Liquids with a Finite Number of Beads per Ring-Polymer

The PEL formalism provides a formal expression for the canonical partition function of a quantum liquid, *Q*(*N*, *V*, *T*).^12^ In the case of a quantum liquid composed of *N* atoms, *Q*(*N*, *V*, *T*) = *T*_*r*_(*ρ̂*), where *T*_*r*_(*ρ̂*) is the trace of the density operator *ρ̂* = exp­(−*βĤ*) and

1 $$Ĥ=\overset{N}{\underset{i=1}{\sum }}\frac{{\mathrm{p̂}}_{i}^{2}}{2m_{i}}+U\left({\mathrm{r̂}}_{1},{\mathrm{r̂}}_{2},...{\mathrm{r̂}}_{N}\right)$$

is the Hamiltonian operator of the system. In eq 1, (**r̂**_*i*_, **p̂**_*i*_) are the position and momentum operators associated with atom *i* = 1, 2, ..., *N*, and *m*_*i*_ is the corresponding mass (*β* = 1/*k*_*B*_ *T* and *k*_*B*_ is the Boltzmann constant).

Using the path-integral formulation of quantum statistical mechanics, one can show that the canonical partition function of the quantum liquid is identical to the canonical partition function of a *classical* system composed of *N* distinguishable ring-polymers, each composed of *n*_*b*_ distinguishable beads, and with peculiar interactions.^23,24^ Specifically,

$$Q\left(N,V,T\right)=\underset{n_{b}⃗\infty }{\lim}\frac{1}{h^{3n_{b}N}}\int_{V}\left[\overset{N}{\underset{i=1}{\prod }}dr_{i}^{1}\cdots dr_{i}^{n_{b}}\right]\int_{-\infty }^{\infty }\left[\overset{N}{\underset{i=1}{\prod }}dp_{i}^{1}\cdots dp_{i}^{n_{b}}\right]\exp\left(-\beta H_{RP}\left(R,P\right)\right)$$ 2

where

3 $$H_{RP}\left(R,P\right)=\overset{N}{\underset{i=1}{\sum }}\overset{n_{b}}{\underset{k=1}{\sum }}\frac{{\left(p_{i}^{k}\right)}^{2}}{2m_{i}^{\prime }}+\overset{N}{\underset{i=1}{\sum }}\overset{n_{b}}{\underset{k=1}{\sum }}\frac{1}{2}k_{sp,i}{\left(r_{i}^{k+1}-r_{i}^{k}\right)}^{2}+\frac{1}{n_{b}}\overset{n_{b}}{\underset{k=1}{\sum }}U\left(r_{1}^{k},r_{2}^{k}...,r_{N}^{k}\right)$$

is the Hamiltonian of the ring-polymer system; *k*_*sp*,*i*_ = *m*_*i*_ *n*_*b*_/(*ℏβ*)^2^ is the (temperature dependent) spring constant of the ring-polymer associated with atom *i* with mass *m*_*i*_; $m_{i}^{\prime }=m_{i}n_{b}$ is the fictitious bead mass. The quantities $\left(r_{i}^{k},p_{i}^{k}\right)$ are the vector position and momentum of the *k*-*th* bead of ring-polymer *i* = 1, 2, ...*N*; $R\equiv \{r_{i}^{k}{\}}_{i=1,2...N;k=1,2...n_{b}}$ and $P\equiv \{p_{i}^{k}{\}}_{i=1,2...N;k=1,2...n_{b}}$ . Note that in eq 3 and throughout this work, $r_{i}^{n_{b}+1}=r_{i}^{1}$ for *i* ∈ {1, 2, ..., *N*}, i.e., the polymers are *ring*-polymers. From eq 3, the potential energy function of the ring-polymer system is given by

4 $$U_{RP}\left(R\right)=\overset{N}{\underset{i=1}{\sum }}\overset{n_{b}}{\underset{k=1}{\sum }}\frac{1}{2}k_{sp,i}{\left(r_{i}^{k+1}-r_{i}^{k}\right)}^{2}+\frac{1}{n_{b}}\overset{n_{b}}{\underset{k=1}{\sum }}U\left(r_{1}^{k},r_{2}^{k}...,r_{N}^{k}\right)$$

It follows from eq 4 that only beads from different ring-polymers with the same label *k* interact with one another. The set of all beads with the same label *k* defines the so-called *k*-th replica of the system, and the term $U\left(r_{1}^{k},r_{2}^{k}...,r_{N}^{k}\right)$ in eq 4 is the total potential energy of replica *k* [the function *U*(···) is defined in eq 1].

Equation 2 provides the exact canonical partition function of the quantum liquid but it requires *n*_*b*_ → ∞. In practice, motivated by PI computer simulations, one chooses a sufficiently large value of *n*_*b*_ for which the properties of the system of interest converge and no longer vary upon further increase in *n*_*b*_. This implies that, *for a given finite (and large) value of n*_*b*_,

5 $$Q\left(N,V,T\right)\approx Q\left(n_{b},N,V,T\right)\equiv \frac{1}{h^{3n_{b}N}}\int_{V}\left[\overset{N}{\underset{i=1}{\prod }}dr_{i}^{1}\cdots dr_{i}^{n_{b}}\right]\int_{-\infty }^{\infty }\left[\overset{N}{\underset{i=1}{\prod }}dp_{i}^{1}\cdots dp_{i}^{n_{b}}\right]\exp\left(-\beta H_{RP}\left(R,P\right)\right)$$

In particular, eq 4 defines the PEL of the ring-polymer system (RP-PEL). It is this RP-PEL which defines the PEL associated with the quantum system of interest. This approach allows one to extend the PEL formalism, originally formulated to study classical systems,^2^ to the case of quantum systems. Within the PEL formalism, and for a given *n*_*b*_, one can show that the partition function of the quantum liquid is given by^2–4,12^

6 $$Q\left(n_{b},N,V,T\right)\approx e^{-\beta \left(E_{IS}(n_{b},N,V,T)-TS_{IS}(n_{b},N,V,T,E_{IS})+F_{vib}(n_{b},N,V,T,E_{IS})\right)}$$

where *E*_*IS*_(*n*_*b*_, *N*, *V*, *T*) is the IS energy defined as the solution of the following equation,

$$1-T{\left(\frac{\partial S_{IS}\left(n_{b},N,V,T,e_{IS}\right)}{\partial e_{IS}}\right)}_{n_{b},N,V,T}+{\left(\frac{\partial F_{vib}\left(n_{b},N,V,T,e_{IS}\right)}{\partial e_{IS}}\right)}_{n_{b},N,V,T}=0\;at\;e_{IS}=E_{IS}$$ 7

In computational studies, *E*_*IS*_ is identified with the average value of the IS energies *e*_*IS*_ sampled by the liquid in equilibrium [at a given (*n*_*b*_, *N*, *V*, *T*)]. In eq 7, *S*_*IS*_(*n*_*b*_, *N*, *V*, *T*, *e*_*IS*_) is the configurational entropy of the system defined as

8 $$S_{IS}\left(n_{b},N,V,T,e_{IS}\right)\equiv k_{B}\ln\left[\Omega_{IS}\left(n_{b},N,V,T,e_{IS}\right)\right]$$

where Ω_*IS*_(*n*_*b*_, *N*, *V*, *T*, *e*_*IS*_) is the number of IS available in the RP-PEL with IS energy *e*_*IS*_ at the given (*n*_*b*_, *N*, *V*, *T*). In addition, in eq 7, *F*_*vib*_(*n*_*b*_, *N*, *V*, *T*, *e*_*IS*_) is the vibrational Helmholtz free energy of the system defined as

9 $$F_{vib}\left(n_{b},N,V,T,e_{IS}\right)\equiv -k_{B}T\ln\left({\langle Q_{l}\left(n_{b},N,V,T\right)\rangle }_{e_{IS}}\right)$$

where *Q*_*l*_(*n*_*b*_, *N*, *V*, *T*) is the partition function of the ring-polymer system when it is constrained to explore only the *l*-th basin of the RP-PEL; ${\langle ...\rangle }_{e_{IS}}$ indicates averaging over all basins *l* in the RP-PEL with IS energy *e*_*IS*_. Specifically,

10 $$Q_{l}\left(n_{b},N,V,T\right)\equiv \frac{1}{h^{3n_{b}N}}\int_{-\infty }^{\infty }\left[\overset{N}{\underset{i=1}{\prod }}dp_{i}^{1}\cdots dp_{i}^{n_{b}}\right]\exp\left(-\beta \overset{N}{\underset{i=1}{\sum }}\overset{n_{b}}{\underset{k=1}{\sum }}{\left(p_{i}^{k}\right)}^{2}/2m_{i}^{\prime }\right)\times \int_{V_{l}}\left[\overset{N}{\underset{i=1}{\prod }}dr_{i}^{1}\cdots dr_{i}^{n_{b}}\right]\exp\left(-\beta \Delta U_{l}\left(R\right)\right)$$

where *V*_*l*_ is the volume in configurational space associated with basin *l*, and $\Delta U_{l}\left(R\right)\equiv U_{RP}\left(R\right)-e_{IS,l}$ is the potential energy of the system in basin *l* relative to its IS energy *e*_*IS*,*l*_.

#### 2.1.1 Harmonic Approximation

An important and useful approximation in the PEL formalism is to assume that the ring-polymer basins are harmonic. It can be shown that, in this harmonic approximation of the PEL, eq 10 can be solved^2,4,12^ resulting in the following expression,

11 $$Q_{l}\left(n_{b},N,V,T\right)=\overset{3N}{\underset{i=1}{\prod }}\overset{n_{b}}{\underset{j=1}{\prod }}\left(\frac{1}{\beta \hbar \omega_{ij}}\right)$$

where $\{\omega_{i,j}=\omega_{i,j}\left(n_{b},N,V,T\right){\}}_{i=1,2,...3N;j=1,2,...n_{b}}$ are the ring-polymer normal mode frequencies when the system is at the IS of basin *l*; equivalently, the set $\left\{\omega_{i,j}^{2}\right\}$ are the 3*Nn*_*b*_ eigenvalues of the mass-weighted Hessian matrix of the ring-polymer system (evaluated at the IS of basin *l*). Relevant to this work, it was shown in refs 12,15 that

12 $$\omega_{i,j}^{2}=\frac{\omega_{i,0}^{2}}{n_{b}^{2}}+\frac{1}{{\left(\beta \hbar \right)}^{2}}\left(2-2\cos\left(\frac{2\pi }{n_{b}}j\right)\right)$$

where (*i* = 1, 2, ...3*N*; *j* = 1, 2, ...*n*_*b*_), and the set $\{\omega_{i,0}^{2}=\omega_{i,0}^{2}\left(N,V\right){\}}_{i}$ are the 3*N* eigenvalues of the mass-weighted Hessian matrix of the *classical* liquid counterpart (evaluated at the same IS associated with basin *l*); see refs 12,15 for details. In other words, remarkably, the 3*Nn*_*b*_ normal modes frequencies of the ring-polymer system associated with the quantum liquid of interest are given by the 3*N* normal mode frequencies of the same liquid but in the classical limit (the frequencies {*ω*_*i*,0_}_*i*=1,2,···3*N*_ are obtained from classical MD simulations).

From eqs 9 and 11, one obtains the following expression for *F*_*vib*_ in the harmonic approximation,

$$F_{vib}^{harm}\left(n_{b},N,V,T,e_{IS}\right)=\frac{1}{\beta }\ln\left[{\left\langle \overset{3N}{\underset{i=1}{\prod }}\overset{n_{b}}{\underset{j=1}{\prod }}\left(\beta \hbar \omega_{ij}\right)\right\rangle }_{e_{IS}}\right]$$ 13

To a good approximation, eq 13 is usually re-written in terms of the so-called shape function,^4^

$$S\left(n_{b},N,V,T,e_{IS}\right)\equiv {\left\langle \ln\left(\overset{3N}{\underset{i=1}{\prod }}\overset{n_{b}}{\underset{j=1}{\prod }}\left(\omega_{i,j}/\omega_{0}\right)\right)\right\rangle }_{e_{IS}}$$ 14

where *ω*_0_ is an arbitrary constant that makes the argument of the ln(···) unit-less.^4^ In eq 14, ${\langle ...\rangle }_{e_{IS}}$ indicates an average over all basins of the RP-PEL with energy *e*_*IS*_; in equilibrium, *e*_*IS*_ → *E*_*IS*_. From eqs 13 and 14, it follows that (harmonic approximation)

15 $$F_{vib}^{harm}\left(n_{b},N,V,T,E_{IS}\right)\approx 3Nn_{b}k_{B}T\ln\left(\beta \hbar \omega_{0}\right)+k_{B}TS\left(n_{b},N,V,T,E_{IS}\right)$$

#### 2.1.2 Anharmonic Contributions

The application of the PEL formalism is not limited to liquids with a harmonic PEL; anharmonic contributions can be included.^4,9,13,15,25,26^ In the PEL formalism for quantum liquids with a finite *n*_*b*_, basin anharmonicities are modeled by decomposing the vibrational free energy as

16 $$F_{vib}\left(n_{b},N,V,T,e_{IS}\right)=F_{vib}^{harm}\left(n_{b},N,V,T,e_{IS}\right)+F_{vib}^{anh}\left(n_{b},N,V,T,e_{IS}\right)$$

where

17 $$\beta F_{vib}^{anh}\left(n_{b},N,V,T,e_{IS}\right)={\mathrm{B̃}}_{0}\left(n_{b},N,V,T\right)+{\mathrm{B̃}}_{1}\left(n_{b},N,V,T\right)e_{IS}$$

The coefficients *B̃*_0_ and *B̃*_1_ are typically represented by second- or third-order polynomials in *T* and fitted to quantities calculated from PI simulations. The methodology is described in detail in ref 13 for the LJBMs studied here. For the weakly quantum LJBM with Planck's constant *h* = *h*_1_ (see Section 3), the RP-PEL is harmonic, so *B̃*_0_ = *B̃*_1_ = 0 [Figure S7b of the SI]. For *h* = *h*_4_, the RP-PEL is anharmonic and both coefficients are nonzero [Figure S7a of the Supporting information SI]. For comparison, PI simulations of q-TIP4P/F water indicate that *B̃*_1_ ≈ 0 while *B̃*_0_ is nonzero, consistent with the PEL of water being anharmonic.^16^

From eq 6, one can obtain the Helmholtz free energy of the quantum liquid *F*(*N*, *V*, *T*) = −*k*_*B*_ *T* ln­(*Q*(*N*, *V*, *T*)) and hence, its thermodynamic properties, including the equation of state.^15–17^ Notably, while *F*(*N*, *V*, *T*) and all thermodynamic properties of the quantum liquid are *n*_*b*_-independent, the properties of the RP-PEL are not. Indeed, all the PEL quantities on the right-hand side of eq 6 vary, in principle, with *n*_*b*_. The following natural questions arise, (i) how do the PEL variables, particularly {*E*_*IS*_, *S*_*IS*_, *F*_*vib*_}, vary with *n*_*b*_?, and (ii) do they converge for *n*_*b*_ → ∞? These questions are of practical relevance, for example, to compare results from computational studies performed with different values of *n*_*b*_. They are also of fundamental importance, e.g., to interpret the PEL formalism, including the concepts of IS and basins shape function, when the number of degrees of freedom of the ring-polymer system diverges (*n*_*b*_ → ∞).

### 2.2 The Continuous Limit: Infinite Number of Beads per Ring-Polymer

In this section, we argue that when *n*_*b*_ → ∞: (A) *S*_*IS*_(*n*_*b*_, *N*, *V*, *T*, *e*_*IS*_) becomes identical to the configurational entropy of the classical liquid counterpart, $S_{IS}^{CL}\left(N,V,e_{IS}\right)$ (accessible from classical MD simulations); (B) *E*_*IS*_(*N*, *V*, *T*) converges as well but *not* to the corresponding expression of the classical liquid counterpart, $E_{IS}^{CL}\left(N,V,T\right)$ ; (C) *F*_*vib*_(*n*_*b*_, *N*, *V*, *T*, *e*_*IS*_) also converges; and (D), under the harmonic approximation of the PEL, $F_{vib}\equiv F_{vib}^{harm}$ becomes identical to the Helmholtz free energy of a (3*N*)-dimensional *quantum* harmonic oscillator with the corresponding frequencies given by the normal mode frequencies of the *classical* liquid counterpart, {*ω*_*i*,0_}_*i*=1,2···3*N*_ (accessible from classical MD simulations).

*(A) S*_*IS*_. PI computer simulations of monatomic,^11,12^ atomistic binary mixtures,^13^ and molecular liquids (water)^15,17,18^ indicate that for large values of *n*_*b*_, the ring-polymers associated with these quantum liquids are collapsed at the IS of the RP-PEL, after potential energy minimization (i.e., at the IS, the *n*_*b*_ beads of each ring-polymer are found in the same position in real space). We also performed path-integral computer simulations of liquid He at *T* > 4 K, where particle exchange is not observed,^27,28^ and confirm that even in this extreme case, the ring-polymers associated with the He atoms are collapsed at the corresponding IS. As explained in refs 12,13,15 the collapse of the ring-polymers at the IS implies that there is a bijection between the IS of the ring-polymer system (in the RP-PEL) and the IS of the classical liquid counterpart (in the CL-PEL), where the IS energy is preserved. Therefore, for large *n*_*b*_, the distribution of IS energies in the RP-PEL, Ω_*IS*_(*n*_*b*_, *N*, *V*, *T*, *e*_*IS*_), must converge to the distribution of IS energies in the CL-PEL, $\Omega_{IS}^{CL}\left(N,V,e_{IS}\right)$ . It follows from eq 8 that, for large *n*_*b*_, the configurational entropy of the ring-polymer system/quantum liquid is also *n*_*b*_-independent (and T-independent), and converges to the configurational entropy of the classical liquid counterpart, $S_{IS}\left(n_{b},N,V,T,e_{IS}\right)\to S_{IS}^{CL}\left(N,V,e_{IS}\right)$ .

In particular, if the CL-PEL is Gaussian then the following expressions apply for *n*_*b*_ → ∞,

18 $$\Omega_{IS}\left(n_{b},N,V,T,e_{IS}\right)\to \Omega_{IS}^{CL}\left(N,V,e_{IS}\right)=\frac{1}{\sqrt{2\pi }\sigma }e^{\alpha N}e^{-{\left(e_{IS}-E_{0}\right)}^{2}/2\sigma^{2}}$$

and

19 $$S_{IS}\left(n_{b},N,V,T,e_{IS}\right)\to S_{IS}^{CL}\left(N,V,e_{IS}\right)\approx k_{B}\left[\alpha N-\frac{{\left(E_{IS}-E_{0}\right)}^{2}}{2\sigma^{2}}\right]$$

where *α* = *α*(*V*), *E*_0_ = *E*_0_(*V*), and *σ* = *σ*(*V*) (*N*- and *T*-independent).^13^

In this work, we show that the ring-polymers remain collapsed at the IS for increasing and large values of *n*_*b*_, providing strong support for eqs 18 and 19 in the limit *n*_*b*_ → ∞.

*(B) E*_*IS*_. *E*_*IS*_(*n*_*b*_, *N*, *V*, *T*) is defined by eq 7. Accordingly, if *S*_*IS*_ and *F*_*vib*_ both converge for large *n*_*b*_ then eq 7 becomes *n*_*b*_-independent and hence, so does *E*_*IS*_. As discussed above, *S*_*IS*_(*n*_*b*_, *N*, *V*, *T*, *e*_*IS*_) converges for large *n*_*b*_ (for ring-polymers collapsed at the IS). Therefore, convergence of *F*_*vib*_(*n*_*b*_, *N*, *V*, *T*, *e*_*IS*_) for large *n*_*b*_ implies that *E*_*IS*_ also converges, *E*_*IS*_(*n*_*b*_, *N*, *V*, *T*) → *E*_*IS*_(*N*, *V*, *T*). As we will show below, our PI simulations of quantum LJBMs show that indeed *E*_*IS*_(*n*_*b*_, *N*, *V*, *T*) → *E*_*IS*_(*N*, *V*, *T*) for large *n*_*b*_. This is found for LJBMs that have a harmonic and anharmonic RP-PEL (with Planck’s constant *h* = *h*_1_ and *h* = *h*_4_, respectively; see Section 3). PI simulations of a water-like monatomic liquid^11,12^ also indicate that *E*_*IS*_(*n*_*b*_, *N*, *V*, *T*) converges with increasing *n*_*b*_. In addition, it is possible to prove analytically, at least within the harmonic approximation of the PEL, that *F*_*vib*_ converges with increasing *n*_*b*_ (see point (D) below). Hence, *E*_*IS*_(*n*_*b*_, *N*, *V*, *T*) → *E*_*IS*_(*N*, *V*, *T*) for large *n*_*b*_ in the case of harmonic RP-PEL.

Interestingly, while *E*_*IS*_(*n*_*b*_, *N*, *V*, *T*) → *E*_*IS*_(*N*, *V*, *T*) for large *n*_*b*_, the resulting *E*_*IS*_(*N*, *V*, *T*) of the ring-polymer system/quantum liquid differs from the IS energy of the classical liquid counterpart, $E_{IS}^{CL}\left(N,V,T\right)$ . This is because in eq 7, the *F*_*vib*_ of the quantum and classical liquids are usually different (eq 7 holds for both classical and quantum liquids with an identical expression for *S*_*IS*_(*N*, *V*, *e*_*IS*_)). Therefore, at a given temperature *T* (and given (*N*, *V*)), the classical and quantum liquid (for *n*_*b*_ → ∞) visit, on average, IS located at different depths of the corresponding PEL.

*(C) F*_*vib*_. That *F*_*vib*_(*n*_*b*_, *N*, *V*, *T*) converges to a unique *F*_*vib*_(*N*, *V*, *T*) for large *n*_*b*_ follows from eq 6 and the discussions in points (A) and (B) above. Specifically, from eq 6,

20 $$\begin{array}{ll} & F\left(n_{b},N,V,T\right)\equiv -k_{B}T\ln Q\left(n_{b},N,V,T\right)\\ & =E_{IS}\left(n_{b},N,V,T\right)-TS_{IS}\left(n_{b},N,V,T,E_{IS}\right)+F_{vib}\left(n_{b},N,V,T,E_{IS}\right) \end{array}$$

As discussed in points (A) and (B) above, for *n*_*b*_ → ∞, *E*_*IS*_(*n*_*b*_, *N*, *V*, *T*) → *E*_*IS*_(*N*, *V*, *T*) and $S_{IS}\left(n_{b},N,V,T,E_{IS}\right)\to S_{IS}^{CL}\left(N,V,E_{IS}\right)$ . In addition, since *Q*(*n*_*b*_, *N*, *V*, *T*) → *Q*(*N*, *V*, *T*) for large *n*_*b*_, all thermodynamic properties as well as the free energies of the system must converge for sufficiently large values of *n*_*b*_ [see, e.g., Figure 1a and b]. It follows from eq 20 that *F*_*vib*_(*n*_*b*_, *N*, *V*, *T*; *E*_*IS*_) → *F*_*vib*_(*N*, *V*, *T*; *E*_*IS*_) (converges) as well for large *n*_*b*_.

**Figure 1:**
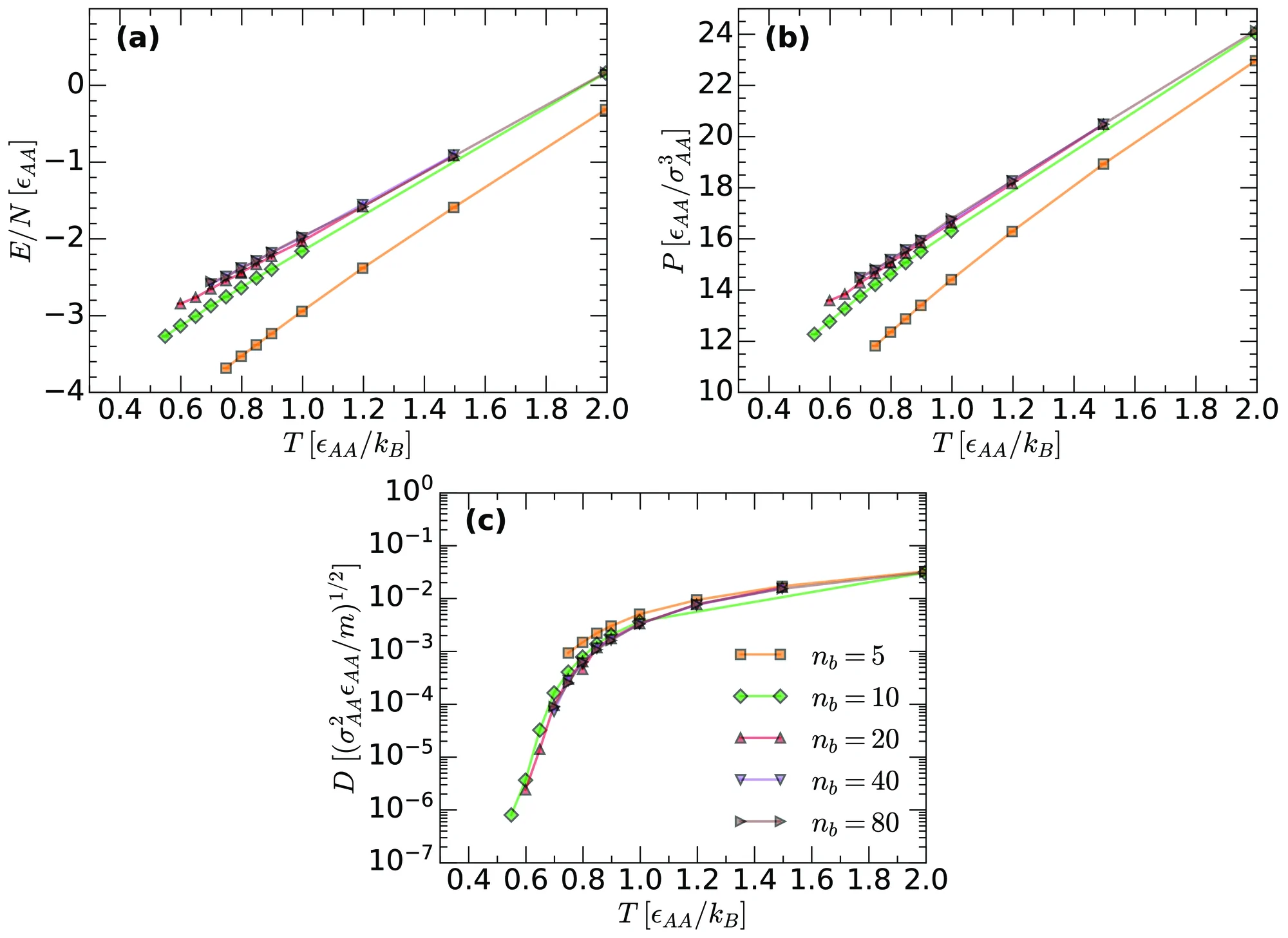
(a) Total energy, (b) pressure, and (c) diffusion coefficient of the (quantum) LJBM studied as a function of temperature. The Planck constant is set to *h* = *h*_4_. All quantities converge for approximately *n_b_* ≥ 20.

*(D) Harmonic approximation*. Equation 13 gives the analytical expression for the vibrational free energy of the ring-polymer system in the harmonic approximation of the PEL, $F_{vib}^{harm}\left(n_{b},N,V,T,e_{IS}\right)$ . Notably, the limit of this expression for *n*_*b*_ → ∞ can be evaluated analytically. To show this, we first note that in eq 11, the product $\overset{n_{b}}{\underset{j=1}{\prod }}\left(1/\beta \hbar \omega_{i,j}\right)$ , with *ω*_*ij*_ given by eq 12, is indeed the canonical partition function of a single *quantum* harmonic oscillator (QHO) with frequency *ω*_*i*,0_. Specifically, this product is the partition function of the QHO evaluated using the path-integral formalism of quantum statistical mechanics, with the particle (oscillator) being represented by a ring-polymer with *n*_*b*_ beads (finite path integral approximation).^23^ It follows that in the limit *n*_*b*_ → ∞, this product must converge to the well-known expression for the canonical partition function of a QHO,

21 $$Q_{QHO}\left(T\right)=\underset{n_{b}\to \infty }{\lim}\overset{n_{b}}{\underset{j=1}{\prod }}\left(\frac{1}{\beta \hbar \omega_{ij}}\right)=\frac{1}{2\sinh\left(\frac{\beta \hbar \omega_{i0}}{2}\right)}$$

Included in the Appendix is a short derivation of eq 21.

Using the identity of eq 21 in eq 11, one can then show from eq 9 that within the harmonic approximation of the RP-PEL, and in the limit *n*_*b*_ → ∞,

$$\begin{array}{ll} F_{vib}^{harm}\left(N,V,T,e_{IS}\right) & =k_{B}T\ln{\left\langle \overset{3N}{\underset{i=1}{\prod }}2\sinh\left(\frac{\beta \hbar \omega_{i,0}}{2}\right)\right\rangle }_{e_{IS}}\\ & \approx k_{B}T{\left\langle \overset{3N}{\underset{i=1}{\sum }}\ln\left(2\sinh\left(\frac{\beta \hbar \omega_{i,0}}{2}\right)\right)\right\rangle }_{e_{IS}} \end{array}$$ 22

The identity in eq 22 is exact within the harmonic approximation of the PEL (the approximation in eq 22 introduces an error of $O\left(N^{1/2}\right)$ , which is irrelevant in the thermodynamic limit; for *h* = *h*_4_, the corresponding error is ∼1%).

The implications of eq 22 are profound. Equation 22 replaces eq 15 in the limit *n*_*b*_ → ∞. In this continuum ring-polymers limit, one can no longer decompose $F_{vib}^{harm}$ into two terms (eq 15), one depending only on *T* and the other containing the relevant information on the PEL topography, quantified by the shape function S . Indeed, the relevance of the shape function fades in the limit *n*_*b*_ → ∞. Equation 22 simplifies the PEL formalism for quantum liquids enormously since it provides an (exact) expression of $F_{vib}^{harm}\left(N,V,T,e_{IS}\right)$ that depends solely on the normal mode frequencies of the classical liquid, {*ω*_*i*,0_}_*i*=1,2..3*N*_, as opposed to the frequencies of the RP-PEL, $\{\omega_{i,j}{\}}_{i=1,2..3N;j=1,2,...n_{b}}$ . In particular, one does not need to evaluate the S any longer, which can be cumbersome (see, e.g., refs 12,15 ). Notably, $S\left(n_{b},N,V,T,e_{IS}\right)$ diverges for *n*_*b*_ → ∞. This follows from eq 15 since $F_{vib}^{harm}\left(N,V,T,e_{IS}\right)$ converges for *n*_*b*_ → ∞ but the term 3*Nn*_*b*_ *k*_*B*_ *T* ln­(*βℏω*_0_) does not.

#### 2.2.1 Anharmonic Contributions

In the continuous ring-polymer limit of the PEL formalism, anharmonic contributions can be treated as explained in Section 2.1.2 for finite *n*_*b*_; the same decomposition also applies within the PEL formalism for classical liquids.^4,9^ Accordingly, eq 16 and eq 17 also appy as *n*_*b*_ → ∞. In eq 16, $F_{vib}^{harm}$ is now given by are given by eq 22. Importantly, since both *F*_*vib*_ and $F_{vib}^{harm}$ converge in the limit *n*_*b*_ → ∞, so does $F_{vib}^{anh}$ . The LJBM with *h* = *h*_1_ is harmonic, so $F_{vib}^{anh}=0$ . Our PI simulations of the LJBM with *h* = *h*_4_ confirm that $F_{vib}^{anh}$ converges as *n*_*b*_ → ∞.

## 3 Computer Simulations Details

We perform classical molecular dynamics (MD) and PI computer simulations of the Lennard-Jones binary mixture (LJBM) defined in ref 29; this is a good glass-former liquid studied extensively in the past.^6,7,30–33^ The system is composed of *N* = 1000 atoms, *N*_*A*_ = 800 type-A and *N*_*B*_ = 200 type-B atoms, with interactions given by the Lennard-Jones pair potential, *V*_*αβ*_(*r*) = 4*ϵ*_*αβ*_[(*σ*_*αβ*_/*r*)^12^ – (*σ*_*αβ*_/*r*)^6^] where *α*, *β* ∈ {*A*, *B*} refer to the atoms type. Following ref 29, we use reduced units with *σ*_*AA*_ = 1.0, *σ*_*BB*_ = 0.80, *σ*_*AB*_ = 0.88 (length), *ϵ*_*AA*_ = 1.0, *ϵ*_*BB*_ = 0.5, *ϵ*_*AB*_ = 1.5 (energy), and *m*_*A*_ = *m*_*B*_ = *m* = 1.0 (mass). The LJ interactions are truncated and shifted at *r*_*c*,*αβ*_ = 2.5*σ*_*αβ*_. We also include a switching function for *r*_*s*,*αβ*_ < *r* < *r*_*c*,*αβ*_ with *r*_*s*,*αβ*_ = 0.9*r*_*c*,*αβ*_, as implemented in the OpenMM software package,^34^ to make the interatomic forces *F*_*αβ*_(*r*) smooth functions of *r*. The simulation box is cubic with periodic boundary conditions along the *x*-, *y*-, and *z*-directions.

To study the LJBM including NQE, we perform ring-polymer molecular dynamics (RPMD) simulations using the path-integral formulation of quantum statistical mechanics (RPMD reduces to PI molecular dynamics when calculating thermodynamic and structural properties). In this framework, each atom of the quantum system is mapped onto a classical ring-polymer with *n*_*b*_ beads.^23,24^ To study atoms delocalization effects (i.e., NQE), we also modify the value of the Planck constant *h*, following refs 11,12,35 . At a given temperature, *h* affects the ring-polymers’ spring constants in eq 3, *k*_*sp*,*i*_ ∝ (*T*/*h*)^2^. Hence, the quantumness of the system increases with increasing *h*, allowing the ring-polymers to expand and enhancing the atoms delocalization; the classical LJBM is recovered for *h* → 0. We consider two values of the Planck constant, *h* = *h*_1_ = 0.2474 and *h* = *h*_4_ = 1.0000, in reduced units of *σ*_*AA*_(*ϵ*_*AA*_ *m*)^1/2^ (see refs 11–13 for details). As shown below (see also ref 13), *h*_1_ denotes the weakly quantum case, while *h*_4_ denotes the more quantum case with stronger atoms delocalization.

The choice of studying two LJBMs, with *h* = *h*_1_ and *h* = *h*_4_, is relevant in the context of the PEL formalism developed here. Specifically, as shown in ref 13, the LJBM with *h* = *h*_1_ has harmonic PEL basins and hence its vibrational free energy is given by eq 22 in the continuous ring-polymer limit. Instead, for *h* = *h*_4_, the PEL basins of the LJBM are not harmonic.^13^ Accordingly, by studying both LJBMs, we compare the PEL formalism in the continuous ring-polymer limit (*n*_*b*_ → ∞) in the absence (*h* = *h*_1_) and presence (*h* = *h*_4_) of basin anharmonicities. Our computer simulation results are qualitatively similar for both LJBMs studied, implying that the PEL formalism in the limit *n*_*b*_ → ∞ is independent of anharmonic contributions. The results for *h* = *h*_4_ are included in the main manuscript; the corresponding results for *h* = *h*_1_ are included in the SI. For *h* = *h*_4_, we run PI simulations with *n*_*b*_ = 5, 10, 20, 40, 80; for *h* = *h*_1_, PI simulations are run for *n*_*b*_ = 10, 20, 40. Classical MD simulations are performed by setting *n*_*b*_ = 1 in the RPMD simulations. The diffusion coefficient *D*(*T*) is computed from the long-time limit of the mean-square displacement of the ring-polymer centroids; see refs 13,15,35–38 for details.

All simulations are performed in the NVT ensemble at density *ρ* = 1.2 (box side length *L* = 9.4 *σ*_*AA*_) over a wide range of temperatures. The temperature is controlled by a local PILE thermostat with stochastic collision frequency *γ* = 0.6. The pressure is evaluated using the estimator given in eq (12.8.43) of ref 23. All simulations are performed using the OpenMM software package (version 7.3.0) modified to include the target Planck constant *h*.^34^ In the OpenMM software package, the masses and LJ parameters are mapped to physical units via *m*_*A*_ = 39.948 amu, *σ*_*AA*_ = 1.0 Å, and *ϵ*_*AA*_ = 1.0 kJ/mol, while all reported quantities remain in reduced units. The time step is *dt* = 0.001 ps for all state points. At each state point, the starting configuration of the ring polymer system/quantum liquid is taken from an independent equilibrium RPMD simulation performed at high temperature, *T* = 5.0. Equilibration runs extend for *t*_*eq*_ ≥ 100 *τ*_*α*_ for most state points. The structural relaxation time *τ*_*α*_ is defined as the time at which the mean-square displacement of type-A particles reaches $1.0{\;\sigma }_{AA}^{2}$ . Production runs extend for *t* ≥ 100 *τ*_*α*_ to obtain a sufficient number of inherent structures (IS).

From the production trajectories, we extract more than 100 independent configurations at each temperature. Two configurations are considered independent if they are separated by at least δ*t* = *τ*_*α*_. For each configuration, we compute the corresponding IS by minimizing the potential energy using the L-BFGS-B algorithm. The minimization is carried out in Python using the *scipy.optimize* routine^39^ with an absolute energy tolerance of 10^–4^; the forces and energies are evaluated in the OpenMM software package using the OpenCL platform with double precision. For each classical IS, we compute the mass-weighted Hessian matrix and diagonalize it to obtain the normal-mode frequencies. The diagonalization is performed using the *NumPy* linear algebra package.^40^

## 4 Results

In this section, we validate the conclusions presented in points (A), (B), (C), and (D) of Section 2.2 using PI computer simulations. The results presented below are based on PI simulations of the (quantum) LJBM described above, where the Planck constant is set to *h* = *h*_4_.^13^ As discussed in ref 13, the RP-PEL of this LJBM (*h* = *h*_4_) is Gaussian but not harmonic. In the SI, we include the results from PI computer simulations for the same LJBM studied here but for *h* = *h*_1_; for this value of the Planck constant, the corresponding RP-PEL is both Gaussian and harmonic.^13^ The conclusions presented in points (A), (B), (C), and (D) of Section 2.2 are in agreement with the PI simulation results for both *h* = *h*_1_ and *h*_4_.

Figure 1 shows the pressure *P*(*T*), total energy *E*(*T*), and diffusion coefficient *D*(*T*) (type-A atoms) of the LJBM obtained from PI computer simulations. Results are for *n*_*b*_ = 5, 10, 20, 40, 80. As expected, all three thermodynamic/dynamical properties converge with increasing *n*_*b*_, and they practically overlap for approximately *n*_*b*_ ≥ 20. Structural properties, such as the radial distribution functions, also converge for *n*_*b*_ ≥ 20.

### 4.1 Ring-Polymers Collapse at the IS and Configurational Entropy

As discussed in point (A) of Section 2.2, if the ring-polymers associated with the atoms in the quantum liquid are collapsed at the IS sampled by the system then $S_{IS}\left(n_{b},N,V,T,e_{IS}\right)\to S_{IS}^{CL}\left(N,V,e_{IS}\right)$ . To show that this is the case for large *n*_*b*_, we calculate the average radius of gyration of the ring-polymers in the instantaneous configurations and corresponding IS (*R*_*g*_(*T*) and *R*_*g*_^*IS*^(*T*), respectively). The average radius of gyration is defined as

23 $$R_{g}^{2}=\frac{1}{N}\left\langle \overset{N}{\underset{i=1}{\sum }}\frac{1}{n_{b}}\overset{n_{b}}{\underset{k=1}{\sum }}{\left({\mathrm{r⃗}}_{i,k}-{\mathrm{r⃗}}_{i,C}\right)}^{2}\right\rangle$$

where *r⃗*_*i*,*k*_ is the vector position of bead *k* = 1, 2, ...*n*_*b*_ of ring-polymer *i* = 1, 2, ...*N*, and *r⃗*_*i*,*C*_ is the position of the centroid (center of mass) of ring-polymer *i*; ⟨···⟩ indicates averaging over time.

Figure 2a shows *R*_*g*_(*T*) (evaluated over all the atoms in the system) as a function of temperature and for different values of *n*_*b*_. Consistent with Figure 1, the values of *R*_*g*_(*T*) converge for *n*_*b*_ ≥ 20. In all cases, and as expected, *R*_*g*_(*T*) increases monotonically upon cooling, implying that the atoms in the LJBM become increasingly delocalized upon cooling. Figure 2b shows *R*_*g*_^*IS*^(*T*). Notably, and consistent with previous PI simulations^12,15^ *R*_*g*_^*IS*^(*T*) ≈ 0 at the IS sampled by the system for all values of *n*_*b*_, i.e., the ring-polymers are collapsed onto single points in real space. Accordingly, it follows from Figure 2b that $S_{IS}\left(n_{b},N,V,T,e_{IS}\right)\to S_{IS}^{CL}\left(N,V,e_{IS}\right)$ for *n*_*b*_ → ∞.

**Figure 2:**
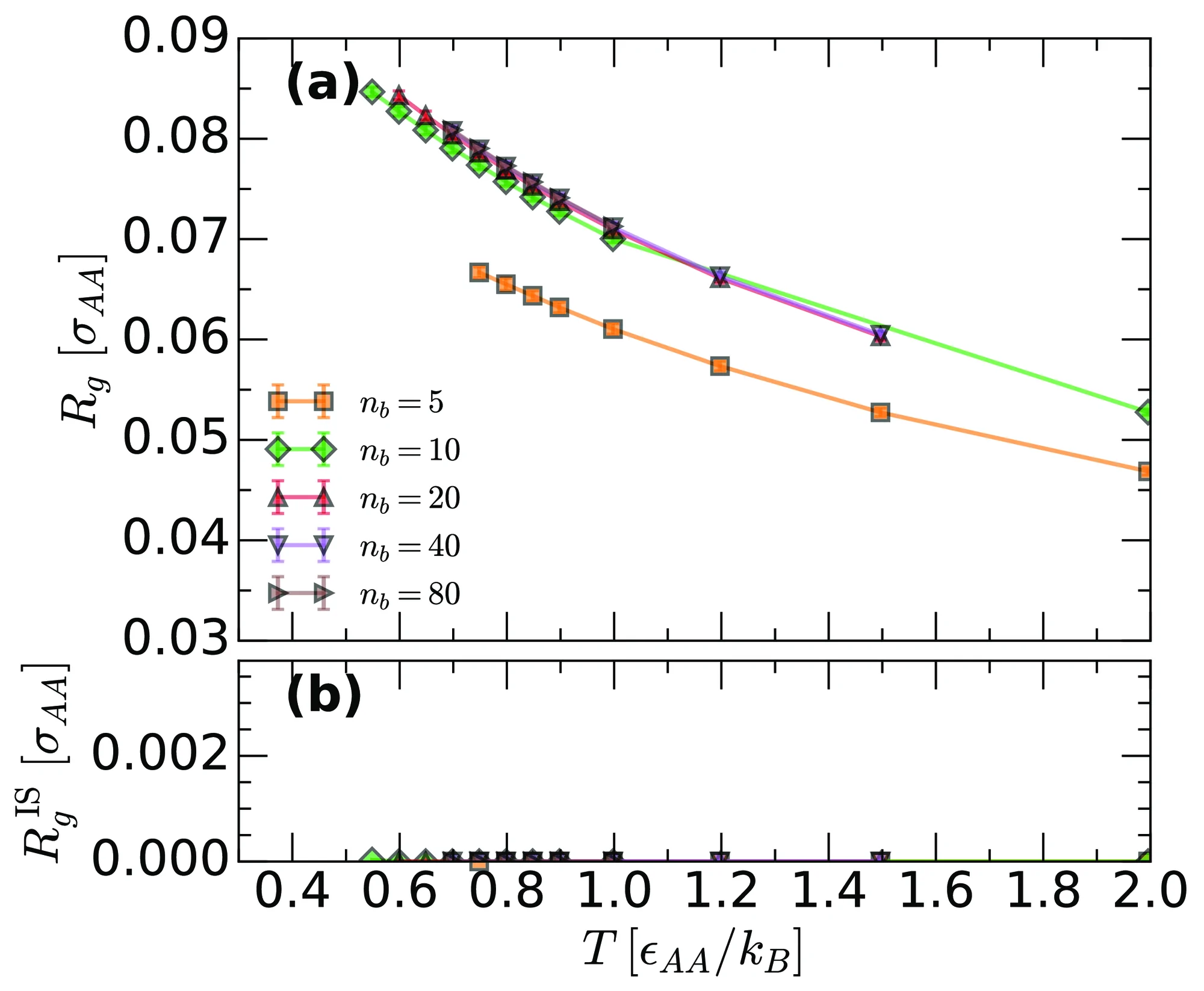
(a) Average radius of gyration *R_g_* (*T*) as a function of temperature for the ring-polymers/atoms in the quantum LJBM studied (*h* = *h*_4_). Results are from PI simulations with *n_b_* = 5, 10, 20, 40, 80 beads per ring-polymer; the values of *R_g_* (*T*) converge for approximately *n_b_* ≥ 20. The monotonic increase of *R_g_* (*T*) with decreasing temperature indicates that atoms in the LJBM become increasingly delocalized upon cooling, as expected. (b) Radius of gyration of the ring-polymers at the IS sampled by the system. *R*_*g,IS*_(*T*) ≈ 0 for all values of *n_b_* and all temperatures studied implying that the ring-polymers collapse to a single point in real space after minimization of the potential energy.

### 4.2 IS Energy

The average energy of the IS sampled by the ring-polymer system *E*_*IS*_ is shown in Figure 3a as a function of temperature and for *n*_*b*_ = 5, 10, 20, 40, 80. Consistent with previous classical and PI simulations,^8,9,11,12,15,19^ *E*_*IS*_(*n*_*b*_, *T*) (*N* and *V* are constants) reaches a plateau at high temperatures, in the PEL-independent regime, and it decreases rapidly upon cooling, in the PEL-influenced regime.^41^ In particular, *E*_*IS*_(*n*_*b*_, *T*) converges to a unique *E*_*IS*_(*T*) for *n*_*b*_ → ∞, consistent with the discussion in point (B) of Section 2.2.

**Figure 3:**
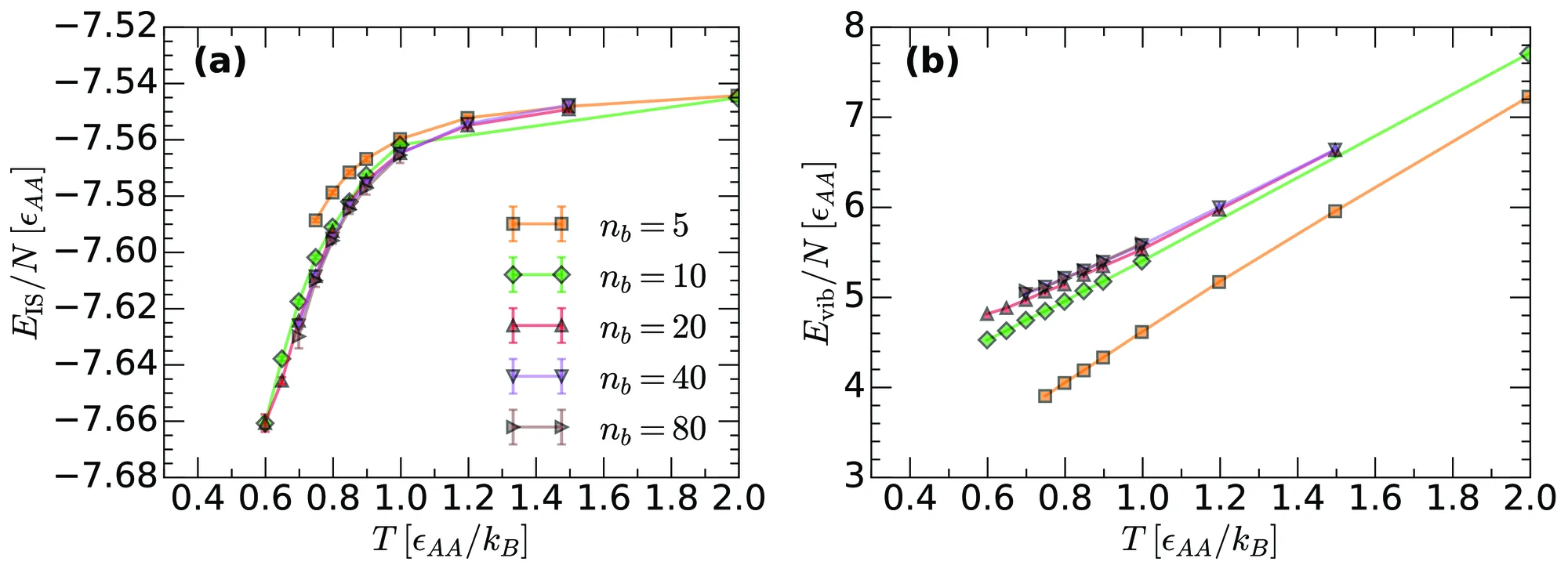
(a) Inherent structure energy *E_IS_* (*T*) and (b) vibrational energy *E_vib_* (*T*) ≡ *E*(*T*) – *E_IS_* (*T*) as a function of temperature for the quantum LJBM with *h* = *h*_4_. Results are for *n_b_* = 5, 10, 20, 40, 80. Both *E_IS_* (*T*) and *E_vib_* (*T*) become *n_b_*-independent (converge) with increasing *n_b_* (see also Section S.IV of the SI).

For comparison, we also include in Figure 3b the vibrational energy, *E*_*vib*_(*n*_*b*_, *T*) ≡ *E*(*n*_*b*_, *T*) – *E*_*IS*_(*n*_*b*_, *T*). *E*_*vib*_ is the contribution to the total energy due to the system exploring the basins of the PEL sampled at a given (*N*, *V*, *T*). Since, *E*(*n*_*b*_, *T*) and *E*_*IS*_(*n*_*b*_, *T*) both converge with increasing *n*_*b*_ [Figure 1a and 3a], so does *E*_*vib*_(*n*_*b*_, *T*). The results presented here are for *h* = *h*_4_, for which the LJBM has an anharmonic RP-PEL.^13^ Indeed, as shown in Figure S7a, the behavior of *E*_*vib*_(*T*) for the LJBM with *h* = *h*_4_ does not follow the corresponding prediction of the PEL formalism within the harmonic approximation [eq S1], i.e., the RP-PEL basins are anharmonic. As shown in the SI, similar results hold for *h* = *h*_1_, for which the RP-PEL of the LJBM is harmonic^13^ [Figure S3 and Section S.IV of the SI]. We also evaluate the average pressure of the system at the sampled IS, *P*_*IS*_(*n*_*b*_, *T*) and the associated vibrational pressure, *P*_*vib*_(*n*_*b*_, *T*) ≡ *P*(*n*_*b*_, *T*) – *P*_*IS*_(*n*_*b*_, *T*). As shown in Figure S5 of the SI, both quantities converge as well for approximately *n*_*b*_ ≥ 20.

### 4.3 Vibrational Helmholtz Free Energy

That *F*_*vib*_(*n*_*b*_, *N*, *V*, *T*; *e*_*IS*_) converges to a well-defined function *F*_*vib*_(*N*, *V*, *T*; *e*_*IS*_) for large *n*_*b*_ is already proven in point (C) of Section 2.2. In this section, we focus on the harmonic approximation of the PEL and present results from PI simulations of the target LJBM (*h* = *h*_4_) showing that $F_{vib}^{harm}\left(n_{b},T\right)$ is given by eq 22 for sufficiently large *n*_*b*_. To show this, we consider the expression for $F_{vib}^{harm}\left(n_{b},N,V,T,e_{IS}\right)$ for finite *n*_*b*_, eq 15, and re-write it as follows,

24 $$\beta F_{vib}^{harm}\left(n_{b},N,V,T,e_{IS}\right)\approx 3Nn_{b}\ln\left(\beta \hbar \omega_{0}\right)+S\left(n_{b},N,V,T,e_{IS}\right)$$

Using eq 24, we evaluate $\beta F_{vib}^{harm}\left(n_{b},e_{IS}\right)$ at a given temperature and for a given value of *n*_*b*_ (*N*, *V* are constant). To do so, we calculate $S\left(n_{b},e_{IS}\right)$ by following the same procedure implemented in refs 12,13,15 . As an example, included in Figure 4a is $\beta F_{vib}^{harm}\left(n_{b},e_{IS}\right)$ as a function of *e*_*IS*_ for *T* = 1.0, obtained using eq 24 with different values of *n*_*b*_. Our PI simulations indicate that, indeed, $F_{vib}^{harm}\left(n_{b},N,V,T,e_{IS}\right)\to F_{vib}^{harm}\left(N,V,T,e_{IS}\right)$ for approximately *n*_*b*_ ≥ 40. Similar conclusions hold for the case *h* = *h*_1_, for which the RP-PEL of the studied LJBM is harmonic; see Figure S4 of the SI.

**Figure 4:**
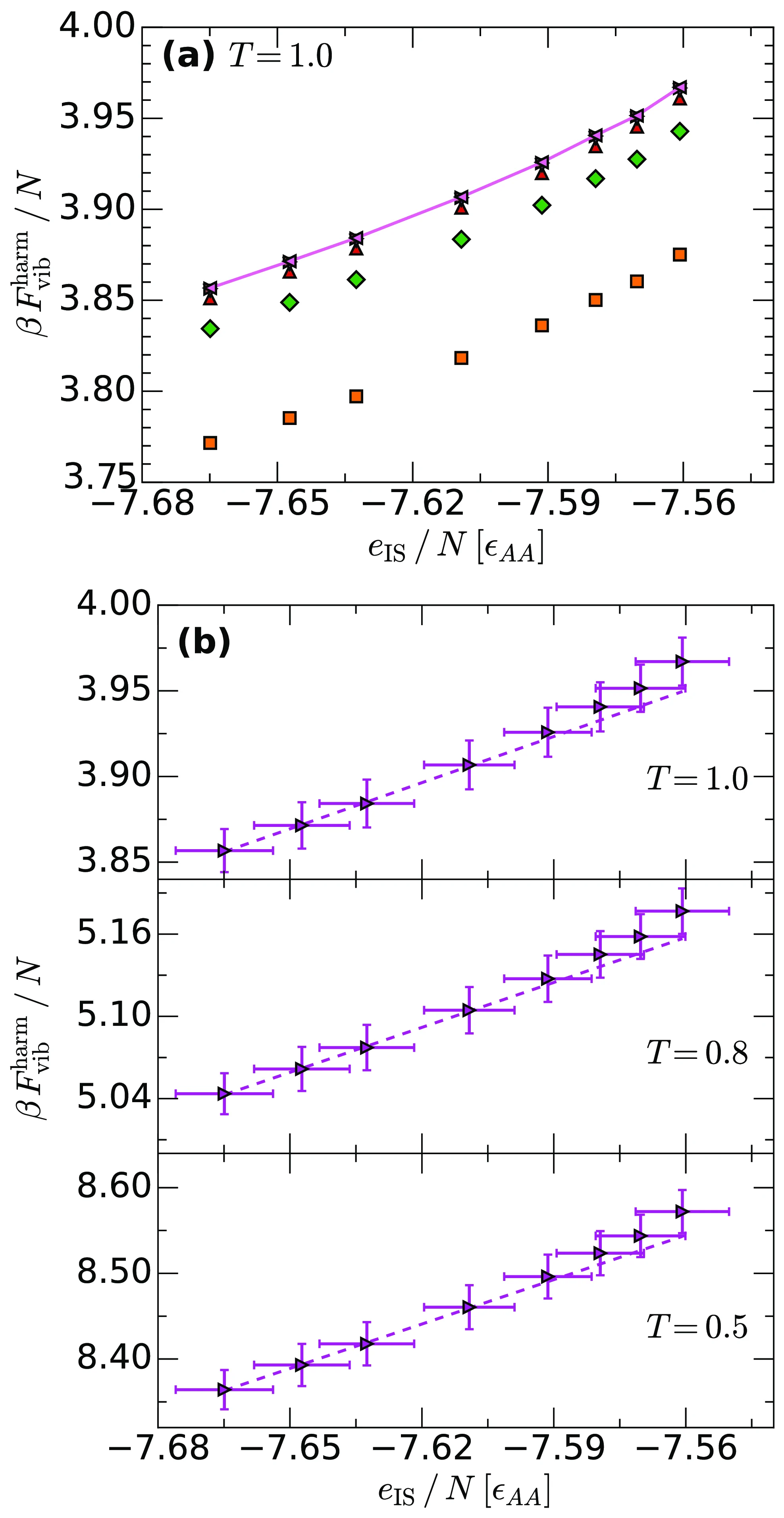
(a) Harmonic contribution to the vibrational Helmholtz free energy of the LJBM (*h* = *h*_4_) as a function of *e_IS_* at *T* = 1.0 and for *n_b_* = 5, 10, 20, 40, 80 (bottom-to-top). For each finite value of *n_b_*, $\beta F_{vib}^{harm}\left(T,e_{IS}\right)$ is evaluated using eq 24 (symbols); see ref 13 for details. $F_{vib}^{harm}\left(n_{b},e_{IS}\right)$ converges for approximately *n_b_* ≥ 40 to the expression given by the (revisited) PEL formalism in the limit *n_b_* → ∞, eq 22 (magenta symbols and interpolating line). Similar results hold at other temperatures, and for the LJBM with *h* = *h*_1_ (see SI). (b) Converged values of $\beta F_{vib}^{harm}\left(T,e_{IS}\right)$ as a function of *e_IS_* obtained using eq 22 and for selected temperatures (symbols with error bars). In all cases, $\beta F_{vib}^{harm}\left(T,e_{IS}\right)$ obeys eq 25 (dashed lines) to a very good approximation, particularly at *e_IS_* < −7.59. Similar results hold for the LJBM with a Planck constant *h* = *h*_1_ (for which the RP-PEL is Gaussian and harmonic; see Figure S4 of the SI).

Figure 4b shows the values of $\beta F_{vib}^{harm}\left(e_{IS}\right)$ for *n*_*b*_ → ∞ at different temperatures obtained from eq 22 (symbols) [the symbols for *T* = 1.0 are the same as the magenta left-triangles in Figure 4a]. Notably, in all cases, and within error bars, we find that

25 $$\beta F_{vib}^{harm}\left(N,V,T,e_{IS}\right)\approx ã\left(N,V,T\right)+\mathrm{b̃}\left(N,V,T\right)e_{IS}$$

­(see dashed lines); *ã*(*N*, *V*, *T*) and *b̃*(*N*, *V*, *T*) are shown in Figure S6 of the SI. Equation 25 holds particularly well at (low) temperatures at which the system explores the low regions of the RP-PEL (approximately, *e*_*IS*_ < −7.59). Equation 25 is analogous to the linear relationship between S and *e*_*IS*_ found in most PEL studies of classical and quantum liquids based on a finite value for *n*_*b*_. Specifically, in the case of quantum liquids, including LJBMs and water, it is found that (finite *n*_*b*_)­

26 $$S\left(n_{b},N,V,T,e_{IS}\right)\approx a\left(n_{b},N,V,T\right)+b\left(n_{b},N,V,T\right)e_{IS}$$

As discussed below, the PEL-variable *b̃*, for *n*_*b*_ → ∞, plays the role of the PEL-variable *b*, for finite *n*_*b*_.

## 5 Summary and Discussion

The main results of this work are stated in points (A), (B), (C), and (D) of Section 2.2, which we validate using PI computer simulations of two LJBMs: one with Planck constant *h* = *h*_4_ (anharmonic RP-PEL; main manuscript) and another with *h* = *h*_1_ (harmonic RP-PEL; Section S.I of the SI). Point (A) states that the ring-polymers associated with the atoms of the quantum liquid collapse to single points in real space when the system is brought to an IS of the RP-PEL. This implies that the configurational entropy converges to that of the classical liquid counterpart as *n*_*b*_ → ∞, i.e., $S_{IS}\left(n_{b},N,V,T,e_{IS}\right)\to S_{IS}^{CL}\left(N,V,e_{IS}\right)$ . Point (B) states that the IS energy becomes *n*_*b*_-independent for large *n*_*b*_; specifically, $E_{IS}\left(n_{b},N,V,T\right)\to E_{IS}\left(N,V,T\right)\neq E_{IS}^{CL}\left(N,V,T\right)$ . Point (C) states that the vibrational contribution to the Helmholtz free energy also converges for large *n*_*b*_, *F*_*vib*_(*n*_*b*_, *N*, *V*, *T*, *e*_*IS*_) → *F*_*vib*_(*N*, *V*, *T*, *e*_*IS*_). In addition [point (D)], for a harmonic RP-PEL, $F_{vib}\equiv F_{vib}^{harm}\left(N,V,T,e_{IS}\right)$ is given by eq 22, which is the limit of eq 15 (applicable for finite *n*_*b*_) as *n*_*b*_ → ∞. Since *F*_*vib*_ and $F_{vib}^{harm}$ converge as *n*_*b*_ → ∞, it follows that $F_{vib}^{anh}$ must converge as well. Consistently, Figure S8 of the SI shows that $E_{vib}^{anh}\left(n_{b},T\right)=E_{vib}\left(n_{b},T\right)-E_{vib}^{harm}\left(n_{b},T\right)$ converges with increasing *n*_*b*_ for *n*_*b*_ = 5, 10, 20, 40, 80 and *h* = *h*_4_. As explained next, these results have important implications for the PEL formalism for quantum liquids and glasses, and further simplify its application in computational studies, particularly when the RP-PEL is harmonic.

1 *The PEL formalism becomes n*_*b*_-*independent*. The PEL formalism developed previously,^12,13,15^ based on an arbitrary (but large) finite value of *n*_*b*_, has the disadvantage that the corresponding PEL properties evaluated using PI simulations can be *n*_*b*_-dependent. This is problematic when comparing studies performed with different values of *n*_*b*_. Our work shows that in the continuous ring-polymer limit (*n*_*b*_ → ∞), the RP-PEL is uniquely defined. In particular, the main PEL properties converge and hence are *n*_*b*_-independent.
2 *No need to evaluate the basin shape function*, S . In the PEL formalism for quantum liquids developed previously, using a finite value of *n*_*b*_, one needs to evaluate the basin shape function S [eq 15]. This applies independently of whether the RP-PEL basins are harmonic or anharmonic. In addition, for practical purposes, one needs to fit the shape function using eq 26, which can be cumbersome.^12,15^ In the continuous ring-polymer limit of the PEL formalism, this is no longer needed. In this limit, eq 15 is replaced by the analytical expression in eq 22, which corresponds to the Helmholtz free energy of a multidimensional quantum harmonic oscillator. To clarify this point, we compare below the typical steps followed in applying the PEL formalism with finite *n*_*b*_ and with *n*_*b*_ → ∞. The continuous-limit formalism developed here is an alternative to, and is not in contradiction with, the previous formalism based on a finite value of *n*_*b*_.
  The procedure employed previously^11–13,15^ to calculate *F*(*N*, *V*, *T*) for a finite *n*_*b*_ can be summarized as follows (for given *N* and *V*):
  (i) Evaluate the shape function $S\left(T,e_{IS}\right)$ using eq 14, and use eq 26 to obtain the PEL variables *a*(*T*) and *b*(*T*); this step also provides $F_{vib}^{harm}\left(T,e_{IS}\right)$ [eq 15].
  (ii) Evaluate *E*_*IS*_(*T*) and obtain the PEL variables *E*_0_ and *σ* ^2^ using eq 28, with *b̃*(*T*) replaced by *b*(*T*).
  (iii) Evaluate the IS entropy *S*_*IS*_(*e*_*IS*_) and *α*(*V*) by calculating the total entropy *S*(*T*) using thermodynamic integration; see refs 2,4,5,12 .
  (iv) Evaluate numerically, using computer simulations, the anharmonic contribution to the free energy, $F_{vib}^{anh}\left(T,e_{IS}\right)$ ; see refs 13,15 .
    For the continuous-limit approach (*n*_*b*_ → ∞), step (i) is replaced by
    (i’) Evaluate $F_{vib}^{harm}\left(T,e_{IS}\right)$ using eq 22, and obtain the PEL variables *ã*(*T*) and *b̃*(*T*). Then continue with steps (ii)–(iv), using *b̃*(*T*) in step (ii).
3 *Harmonic quantum liquids and glasses: Avoiding path-integral computer simulations*. For quantum liquids with harmonic RP-PEL basins, the Helmholtz free energy is given by eq 20, which can be rewritten as follows:

27 $$F\left(N,V,T\right)=E_{IS}\left(N,V,T\right)-TS_{IS}^{CL}\left(N,V,E_{IS}\right)+F_{vib}^{harm}\left(N,V,T,E_{IS}\right)$$

where $F_{vib}^{harm}\left(N,V,T,E_{IS}\right)$ is given by [Disp-formula eq22]. If the RP-PEL is also Gaussian, then $S_{IS}^{CL}\left(N,V,E_{IS}\right)$ is given by eq 19 and eq 7 gives the average IS energy,^12,15^

28 $$E_{IS}\left(N,V,T\right)=E_{0}\left(V\right)-\sigma^{2}\left(V\right)\left(\beta +\mathrm{b̃}\left(N,V,T\right)\right)$$

where $\mathrm{b̃}\left(N,V,T\right)\equiv {\left(\partial F_{vib}^{harm}\left(N,V,T,e_{IS}\right)/\partial e_{IS}\right)}_{N,V,T}$ (*e*_*IS*_ → *E*_*IS*_ in equilibrium); see also eq 25. For the Gaussian case, it follows that *F*(*N*, *V*, *T*) depends only on the PEL variables {*α*(*V*), *E*_0_(*V*), *σ* ^2^(*V*)} and the (classical) normal mode frequencies {*ω*_*i*,0_}_*i*=1,2,···3*N*_ which are all accessible in classical MD simulations. Therefore, for Gaussian and harmonic RP-PELs, one can obtain an analytical expression for the Helmholtz free energy of the *quantum* system using *classical* computer simulations, avoiding the more expensive PI computer simulations. The Gaussian approximation is not essential here, although it applies to most liquids studied within the PEL framework,^2,4,5^ including water.^9,10,22^ If the Gaussian approximation does not hold, eq 28 is replaced by the corresponding expression resulting from eq 7.

The cases of non-annealing glasses and crystals are particularly interesting. As discussed in ref 42, for non-annealing glasses and crystals, *S*_*IS*_ plays a secondary role. In particular, the Gaussian approximation is not required. For example, for atomic crystals and non-annealing glasses *S*_*IS*_ = 0, while for water *S*_*IS*_ = *S*_*IS*_(*N*).^42^ Moreover, the PEL basins of crystals and non-annealing glasses can be considered to be harmonic at low temperatures. Therefore, for low temperature crystals and glasses, eq 27 reduces to

29 $$F\left(N,V,T\right)=E_{IS}^{0}\left(N,V\right)+TS_{IS}^{0}\left(N\right)+F_{vib}^{harm}\left(N,V,T,E_{IS}\right)$$

where $E_{IS}^{0}\left(N,V\right)$ and $S_{IS}^{0}\left(N\right)$ depend on the specific crystal/non-annealing glass considered, and $F_{vib}^{harm}\left(N,V,T,E_{IS}\right)$ is given by eq 22. Notably, the resulting free energy predicted by the PEL formalism, eq 29, is nothing else but the free energy of quantum ice and amorphous ice under the so-called *quasi-harmonic approximation* (QHA).^43,44^ The QHA was validated for different ices using PI simulations in refs 45,46 .

Most of the discussion above for quantum liquids with a harmonic RP-PEL also applies if the RP-PEL is anharmonic. However, in this case, one needs to include anharmonicities in *F*_*vib*_. Specifically, in the limit *n*_*b*_ → ∞, and in the presence of basin anharmonicities, the vibrational free energy the vibrational free energy is given by eq 16 where, $F_{vib}^{harm}\left(N,V,T,e_{IS}\right)$ is given by eq 22. As explained in detail in refs 13,15 $F_{vib}^{anh}\left(N,V,T,e_{IS}\right)$ can be evaluated numerically using PI computer simulations (see Sections 2.1.2 and 2.2.1, and refs 13,15 ). An important implication of including the effects due to the RP-PEL basin anharmonicities is that in principle, it is no longer possible to obtain *F*(*N*, *V*, *T*) solely from classical MD simulations, as is the case for quantum liquids with a harmonic RP-PEL.

We conclude with a brief discussion of the general applicability of the PEL formalism in the continuous ring-polymer limit and potential applications. The PEL formalism can be applied to describe the thermodynamic behavior of classical or quantum liquids independently of whether the corresponding PEL is harmonic or anharmonic. The practical power of the PEL formalism is to provide analytical expressions for the liquid’s free energy based on a handful (six to eight) PEL quantities that need to be obtained from MD/PI computer simulations. These PEL quantities are inherent to the system of interest and hence, need to be sampled from MD/PI simulations. Notably, the MD/PI simulations can be performed at relatively high temperatures, where computational times are accessible. The PEL expressions obtained at such temperatures can then be used to predict the equation of state (phase diagram) of the liquid of interest at very low temperatures, where MD/PI simulations become computationally expensive or inaccessible.

Perhaps one of the most important practical applications of the PEL formalism has been to evaluate the *F*(*N*, *V*, *T*) of supercooled water using computer simulations and to locate water’s liquid–liquid critical point (LLCP). Classical MD simulations, complemented with the PEL formalism for classical liquids, have been used successfully to locate the LLCP in different classical water models, including the empirical SPCE,^8^ TIP4*P*/2005,^9^ and q-TIP4P/F,^10^ as well as the machine-learned DP-MBpol model.^17,22^ In all these cases, the CL-PEL is Gaussian but anharmonic. Similarly, PI simulations, combined with the PEL formalism for quantum liquids using a finite value of *n*_*b*_ [following steps (i)–(iv) above], have been used to study the phase behavior of supercooled water, including the location of its LLCP.^15–17^ It would be important in the future to test the approach presented here for *n*_*b*_ → ∞ [steps (i’), (ii)–(iv)] to estimate the location of the LLCP in different water models, including models derived from first-principles calculations. Preliminary estimations based on the empirical q-TIP4P/F water model indicate that this is possible, but one needs to perform extensive PI simulations with $n_{b}\approx O\left(100\right)$ . Our preliminary results for q-TIP4P/F water are consistent with the main results presented in this work [points (A)–(D) of Section 2.2]. The fact that the PEL formalism also works for machine-learned potentials^22^ opens the door for its application to the study of molecular liquids other than water, with models that include quantum accuracy and where NQE are relevant.^47^ The PEL formalism for quantum liquids presented in this work can be applied to atomistic and molecular liquids where NQE are relevant and where particle exchange can be neglected. In some cases, such as ^4^He at very low temperatures, quantum statistics may play a relevant role. It would be interesting in the future to explore the possibility to include particle-exchange in the PEL formalism. Another interesting future direction could be to extend the PEL formalism to systems where nonadiabatic effects, including geometric-phase effects associated with conical intersections, may be relevant.^48^

## Supplementary Material





## Data Availability

The data underlying this study are available in the published article and the Supporting Information. The simulation details and parameters needed to reproduce the calculations are described in the manuscript. The simulations were performed with OpenMM version 7.3.0, modified to include the target Planck constant as described in the manuscript, and the analyses used SciPy and NumPy. OpenMM is available at https://openmm.org/. The numerical data underlying Figures 1–4 and S1–S8, OpenMM input files, and one sample starting configuration for each of the LJBM and FJ systems are provided as Supporting Information.

## Appendix

### Canonical Partition Function of a Quantum Harmonic Oscillator Using Path-Integrals

Here we present a brief derivation for the identity in eq 21. Using the path-integral formulation of quantum statistical mechanics, one can express the canonical partition function of a one-dimensional quantum harmonic oscillator (QHO) as follows,^23,50^

A1
$$Q_{QHO}\left(T\right)=\underset{n_{b}\to \infty }{\lim}Q_{QHO}^{\left(n_{b}\right)}\left(T\right)$$

where

A2
$$Q_{QHO}^{\left(n_{b}\right)}\left(T\right)={\left(\frac{mn_{b}}{2\pi \beta \hbar^{2}}\right)}^{n_{b}/2}\int \left[\overset{n_{b}}{\underset{k=1}{\prod }}dx_{k}\right]\exp\left[-\beta \overset{n_{b}}{\underset{k=1}{\sum }}\left(\frac{m\omega_{0}^{2}}{2n_{b}}x_{k}^{2}+\frac{mn_{b}}{2\beta^{2}\hbar^{2}}{\left(x_{k+1}-x_{k}\right)}^{2}\right)\right]$$

This expression follows from eqs 2 and 3 for the case *N* = 1 and $U=U\left(\mathrm{x̂}\right)=\frac{1}{2}m\omega_{0}^{2}{\left(\mathrm{x̂}\right)}^{2}$ . In eq A2, *m* is the particle mass, *ω*_0_ is the natural frequency of the harmonic oscillator, and $x_{n_{b}+1}=x_{1}$ . Briefly, *Q*_*QHO*_(*T*) is identical to the partition function of a classical ring-polymer with spring constant $k_{sp}=\frac{mn_{b}}{\beta^{2}\hbar^{2}}$ , beads mass *m*′ = *mn*_*b*_, subjected to a harmonic potential with frequency $\omega_{0}^{\prime }=\omega_{0}/\sqrt{n_{b}}$ ; $\left\{x_{1},x_{2},...x_{n_{b}}\right\}$ are the beads coordinates.

The argument in the exponential of eq A2 is a quadratic form and can be written as

A3
$$\phi \equiv -\frac{1}{2}x^{T}Ax$$

where the column vector $x={(x_{1}x_{2}...x_{n_{b}})}^{T}$ and

A4
$$A={\left(\begin{array}{llllll} c & d & 0 & \cdots & 0 & d\\ d & c & d & \ddots & & 0\\ 0 & d & c & \ddots & \ddots & \vdots \\ \vdots & \ddots & \ddots & \ddots & d & 0\\ 0 & & \ddots & d & c & d\\ d & 0 & \cdots & 0 & d & c \end{array}\right)}_{n_{b}\times n_{b}}$$

where $c=\beta m\omega_{0}^{2}/n_{b}+2mn_{b}/\left(\beta \hbar^{2}\right),d=-mn_{b}/\left(\beta \hbar^{2}\right)$ . Since **A** is real and symmetric, it can be diagonalized by a unitary matrix **V** ^51^ as $Ã=V^{†}AV=\mathrm{diag}\left(\lambda_{1},...,\lambda_{n_{b}}\right)$ where $\lambda_{j}=\beta m\omega_{0}^{2}/n_{b}+\left(2mn_{b}/\beta \hbar^{2}\right)\left(1-\cos\left(2\pi j/n_{b}\right)\right)$ (*j* = 1, ..., *n*_*b*_) are the eigenvalues of **A**.^51^ We introduce the following change of variables,

A5
$$x=V\mathrm{x̃},,\mathrm{x̃}=V^{-1}x=V^{†}x$$

It follows from eqs 32 and 34 that

A6
$$\phi =-\frac{1}{2}{\mathrm{x̃}}^{T}Ã\mathrm{x̃}=-\frac{1}{2}\overset{n_{b}}{\underset{j=1}{\sum }}\lambda_{j}{\mathrm{x̃}}_{j}^{2}$$

Since the transformation matrix **V** is unitary, the volume element in the integral of eq 31 is preserved, implying that

A7
$$Q_{QHO}^{\left(n_{b}\right)}\left(T\right)={\left(\frac{mn_{b}}{2\pi \beta \hbar^{2}}\right)}^{n_{b}/2}\int \left[\overset{n_{b}}{\underset{j=1}{\prod }}d{\mathrm{x̃}}_{j}\right]\exp\left[-\beta \overset{n_{b}}{\underset{j=1}{\sum }}\left(\frac{1}{2}m^{\prime }\omega_{j}^{2}{\mathrm{x̃}}_{j}^{2}\right)\right]$$

where

A8
$$\omega_{j}^{2}\equiv \lambda_{j}/\left(mn_{b}\beta \right)=\frac{\omega_{0}^{2}}{n_{b}^{2}}+\frac{1}{{\left(\beta \hbar \right)}^{2}}\left[2-2\cos\left(\frac{2\pi j}{n_{b}}\right)\right],,j=1,...,n_{b}$$

Equation 36 is the partition function of *n*_*b*_ independent harmonic oscillators with frequencies $\{\omega_{j}{\}}_{j=1,2,...n_{b}}$ . This is an *n*_*b*_-dimensional Gaussian integral which can be solved, resulting in

A9
$$Q_{QHO}^{\left(n_{b}\right)}\left(T\right)=\overset{n_{b}}{\underset{j=1}{\prod }}{\left(\beta \hbar \omega_{j}\right)}^{-1}$$

To evaluate the product in eq 38 in the limit *n*_*b*_ → ∞, we define *x*_0_ ≡ *βℏω*_0_ and introduce the auxiliary constant $\theta_{n_{b}}>0$ ,

A10
$$\cosh\theta_{n_{b}}=1+\frac{x_{0}^{2}}{2n_{b}^{2}}$$

It follows from eqs 37 and 39 that

$$\beta \hbar \omega_{j}={\left[2\cosh\theta_{n_{b}}-2\cos\left(\frac{2\pi j}{n_{b}}\right)\right]}^{1/2}$$
A11

As shown in ref 50 (Section 2.7, eq (2.68) and the expression below it), the following identity holds,

A12
$$\overset{n_{b}}{\underset{j=1}{\prod }}\left[2\cosh\theta_{n_{b}}-2\cos\left(\frac{2\pi j}{n_{b}}\right)\right]=2\left[\cosh\left(n_{b}\theta_{n_{b}}\right)-1\right]$$

Therefore,

A13
$$\overset{n_{b}}{\underset{j=1}{\prod }}\left(\beta \hbar \omega_{j}\right)=\sqrt{2\left[\cosh\left(n_{b}\theta_{n_{b}}\right)-1\right]}=2\sinh\left(\frac{n_{b}\theta_{n_{b}}}{2}\right)$$

where the last term follows from the identity cosh­(2*α*) = 1 + 2sinh^2^(*α*). To evaluate this expression in the limit *n*_*b*_ → ∞, we use the Taylor expansion for $\theta_{n_{b}}$ ,

A14
$$\theta_{n_{b}}=\mathrm{arcosh}\left(1+\frac{x_{0}^{2}}{2n_{b}^{2}}\right)=\frac{x_{0}}{n_{b}}+O\left(n_{b}^{-3}\right)$$

Hence, $n_{b}\theta_{n_{b}}\to x_{0}$ for *n*_*b*_ → ∞. Using this expression in eq 42, one finally obtains

A15
$$Q_{QHO}\left(T\right)=\underset{n_{b}\to \infty }{\lim}\overset{n_{b}}{\underset{j=1}{\prod }}{\left(\beta \hbar \omega_{j}\right)}^{-1}={[2\sinh(x_{0}/2)]}^{-1}={[2\sinh(\beta \hbar \omega_{0}/2)]}^{-1}$$

Equation 21 follows from eq 44 with the substitution *ω*_0_ ≡ *ω*_*i*,0_ and *ω*_*j*_ ≡ *ω*_*i*,*j*_.

## Notes

Numerical data underlying Figures 1−4 and S1−S8; OpenMM input and reporter files for the LJBM and FJ simulations; and one sample starting configuration for each system (ZIP)
Additional PEL properties of the LJBM obtained from RPMD simulations with *h* = *h*_4_ [specifically, the IS and vibrational pressures as a function of temperature (*P*_*IS*_(*T*) and *P*_*vib*_(*T*)), and the coefficients *ã*(*T*) and *b̃*(*T*) defined in eq 25]; the results for the LJBM obtained from RPMD simulations with *h* = *h*_1_; the RP-PEL of the LJBMs is harmonic for *h* = *h*_1_ and anharmonic for *h* = *h*_4_ (PDF)
