# Supplementary material for: The Potential Energy Landscape Formalism for Quantum Liquids in the Continuous Ring-Polymer Limit
Source: J Chem Theory Comput. 2026 Sep 17;22(19):9963–75. doi: 10.1021/acs.jctc.6c01077 (PMC13671181; doi:10.1021/acs.jctc.6c01077)
Supplement: Supplementary file 1 [file ct6c01077_si_002.pdf]

Supporting Information for "The Potential Energy Landscape Formalism for Quantum Liquids in the Continuous Ring-Polymer Limit"

Yang Zhou[1,2,*], Gustavo E. Lopez[3,4,*], and Nicolas Giovambattista[1,2,3*]

[1] *Ph.D. Program in Physics,*
The Graduate Center of the City University of New York,
New York, NY 10016, United States

[2] *Department of Physics,*
Brooklyn College of the City University of New York,
Brooklyn, New York 11210, United States

[3] *Ph.D. Program in Chemistry,*
The Graduate Center of the City University of New York,
New York, NY 10016, United States

[4] *Department of Chemistry,*
Lehman College of the City University of New York,
Bronx, New York 10468, United States

S.I. RESULTS FROM PATH-INTEGRAL COMPUTER SIMULATIONS OF A LENNARD-JONES BINARY MIXTURE WITH SMALL PLANCK'S CONSTANT ($h = h_1$)

The results presented in the main manuscript are obtained from path-integral (PI) computer simulations of a Lennard-Jones binary mixture (LJBM) with Planck's constant $h = h_4 = 1.0000$, for which the nuclear quantum effects (NQE) and atomic delocalization are relevant. In particular, for $h = h_4$, the ring-polymers potential energy landscape (RP-PEL) is *anharmonic* (and Gaussian). In this section, we present results from PI computer simulations for the same LJBM studied in the main manuscript but where the Planck's constant is smaller, $h = h_1 = 0.2474$. This LJBM is weakly quantum, and its properties are close to those found for the corresponding classical LJBM using classical molecular dynamics (MD) simulations [1]. Relevant to this work, for $h = h_1$, the RP-PEL of the LJBM is *harmonic* (and Gaussian). The results are presented in Figs. S1-S4 which correspond, respectively, to Figs. 1-4 of the main manuscript. In the case of $h = h_1$, we only include results for a number of beads per ring-polymer $n_b = 10, 20, 40$. Briefly, all the thermodynamic, structural, dynamical, and PEL properties studied already converge for $n_b \geq 10$. The conclusions presented in the main manuscript for $h = h_4$ (Figs. 1-4) hold as well for $h = h_1$ (Figs. S1-S4).

* yzhou4@gradcenter.cuny.edu, gustavo.lopez1@lehman.cuny.edu, ngiovambattista@brooklyn.cuny.edu

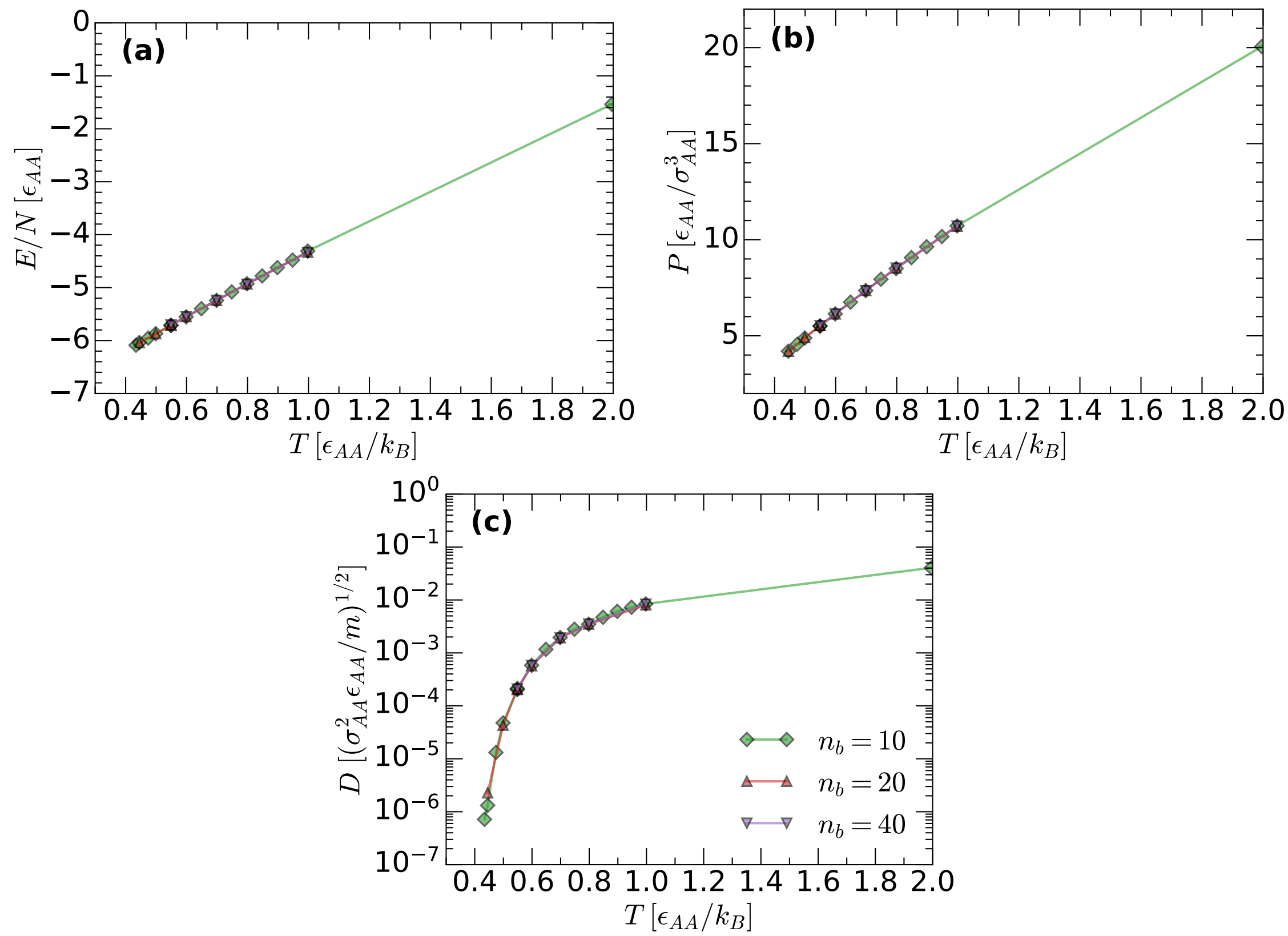

FIG. S1. (a) Total energy, (b) pressure, and (c) diffusion coefficient of the (quantum) LJBM studied as a function of temperature. The Planck constant is set to $h = h_1 = 0.2474$. Results are for $n_b = 10,\ 20,\ 40$, and all quantities converge for $n_b \geq 10$.

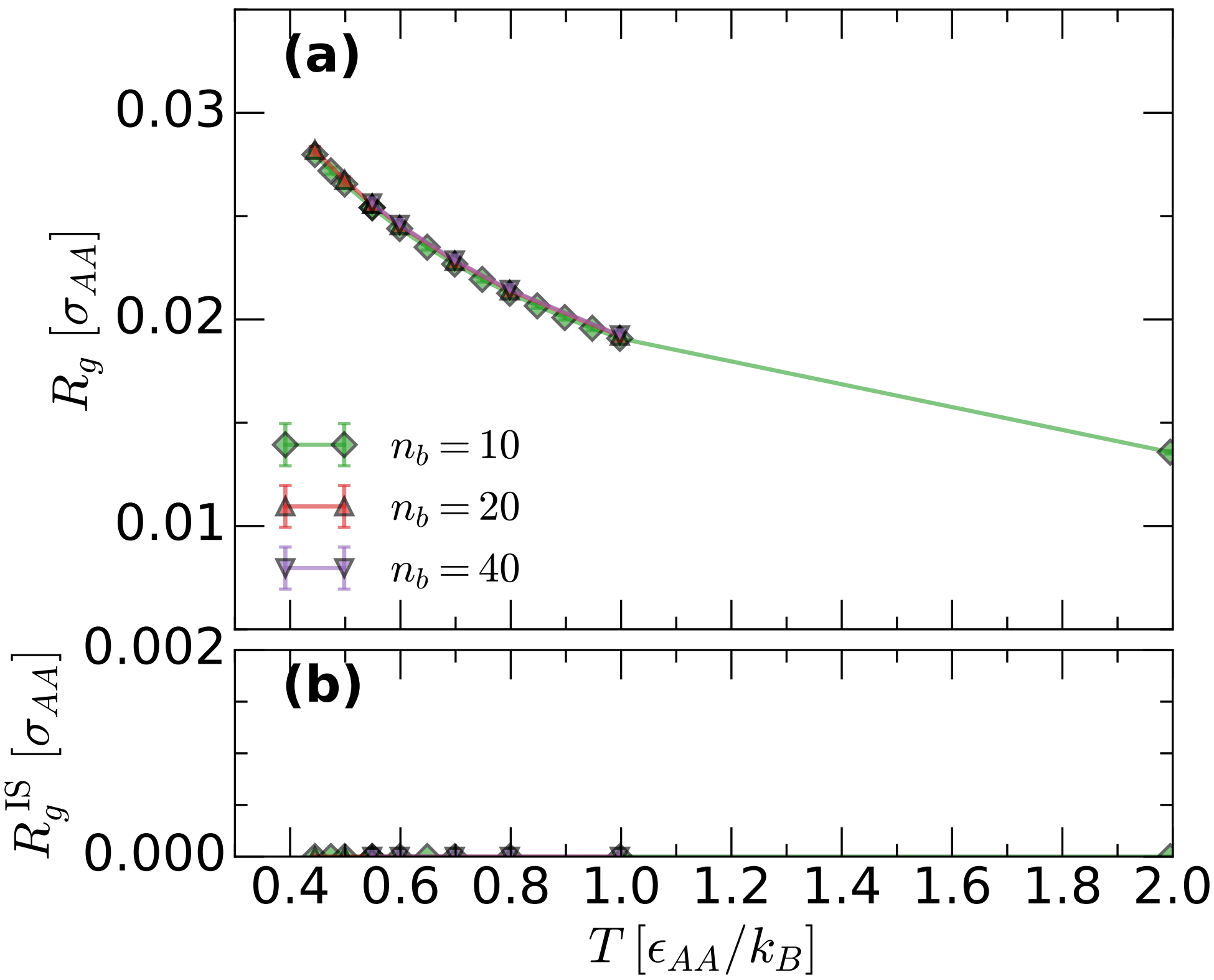

FIG. S2. (a) Average radius of gyration $R_g(T)$ as a function of temperature for the ring-polymers/atoms in the quantum LJBM studied ($h = h_1 = 0.2474$). Results are from PI simulations with $n_b = 10,\ 20,\ 40$ beads per ring-polymer; the values of $R_g(T)$ converge for $n_b \geq 10$. The monotonic increase of $R_g(T)$ with decreasing temperature indicates that atoms in the LJBM become increasingly delocalized upon cooling, as expected. (b) Radius of gyration of the ring-polymers at the IS sampled by the system. $R_{g,IS} \approx 0$ for all values of n_b and all temperatures studied, implying that the ring-polymers collapse to a single point in real space after minimization of the potential energy.

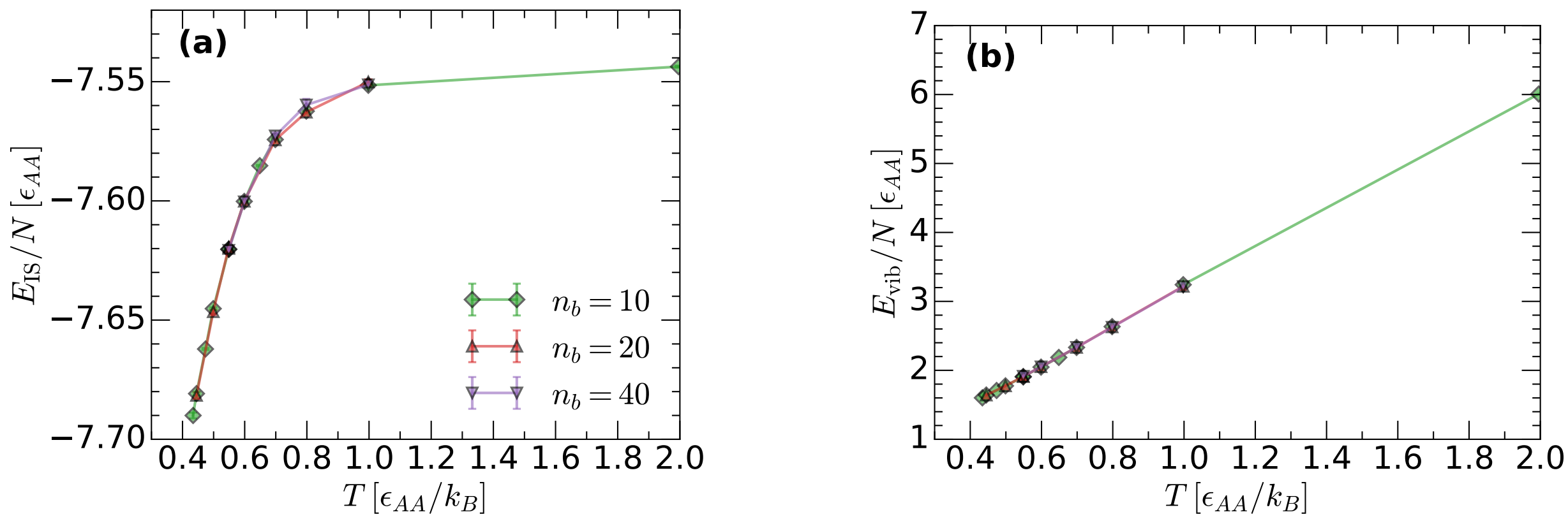

FIG. S3. (a) Inherent structure energy $E_{IS}(T)$ and (b) vibrational energy $E_{vib}(T) \equiv E(T) - E_{IS}(T)$ as a function of temperature for the quantum LJBM with $h = h_1 = 0.2474$. Results are for $n_b = 10,\ 20,\ 40$. Both $E_{IS}(T)$ and $E_{vib}(T)$ become n_b-independent (converge) with increasing n_b.

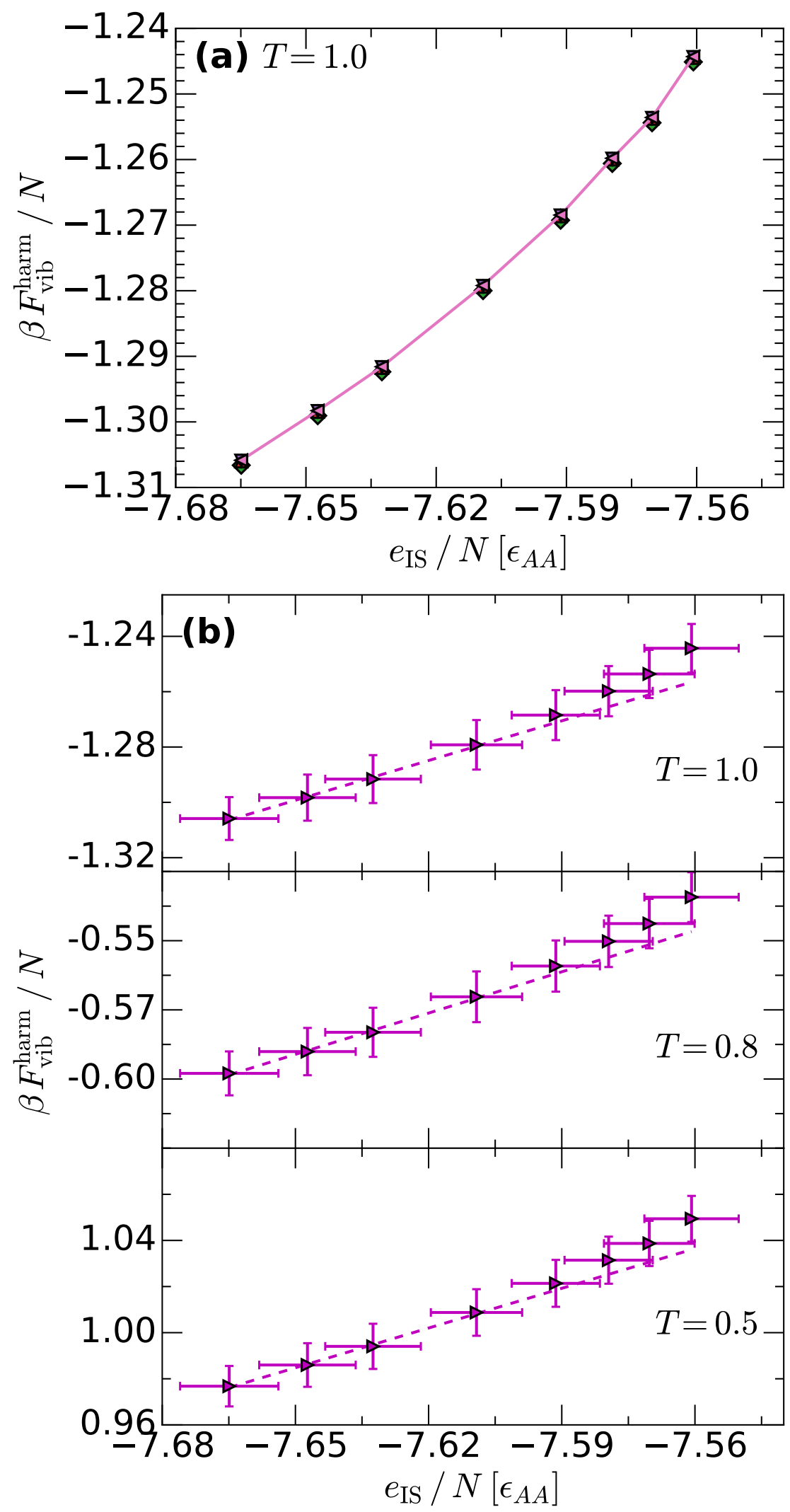

FIG. S4. (a) Harmonic contribution to the vibrational Helmholtz free energy of the LJBM ($h = h_1 = 0.2474$) as a function of e_{IS} at $T = 1.0$ and for $n_b = 10,\ 20,\ 40$. For each finite value of n_b, $\beta F_{vib}^{harm}(T, e_{IS})$ is evaluated using Eq. 24 of the main manuscript (symbols). $F_{vib}^{harm}(n_b, e_{IS})$ converges for $n_b \geq 10$ to the expression given by the revisited PEL formalism in the limit $n_b \to \infty$, Eq. 22 of the main manuscript (magenta symbols and interpolating line). (b) Converged values of $\beta F_{vib}^{harm}(T, e_{IS})$ as a function of e_{IS} obtained using Eq. 22 of the main manuscript and for selected temperatures (symbols with error bars). In all cases, $\beta F_{vib}^{harm}(T, e_{IS})$ obeys Eq. 25 of the main manuscript (dashed lines) to a very good approximation, particularly at $e_{IS} < -7.59$.

S.II. INHERENT STRUCTURES AND VIBRATIONAL PRESSURE

Included in Fig. S5(a)(b) are the IS pressure, $P_{IS}(T)$, and vibrational pressure, $P_{vib}(T) \equiv P(T) - P_{IS}(T)$, of the LJBM studied in the main manuscript with Planck's constant $h = h_4$. As for the case of the IS and vibrational energies (Fig. 3 of the main manuscript), both $P_{IS}(T)$ and $P_{vib}(T)$ converge for $n_b \geq 20$. Similar results hold for the LJBM with $h = h_1$; as shown in Fig. S5(c)(d), $P_{IS}(T)$ and $P_{vib}(T)$ converge for $n_b \geq 10$.

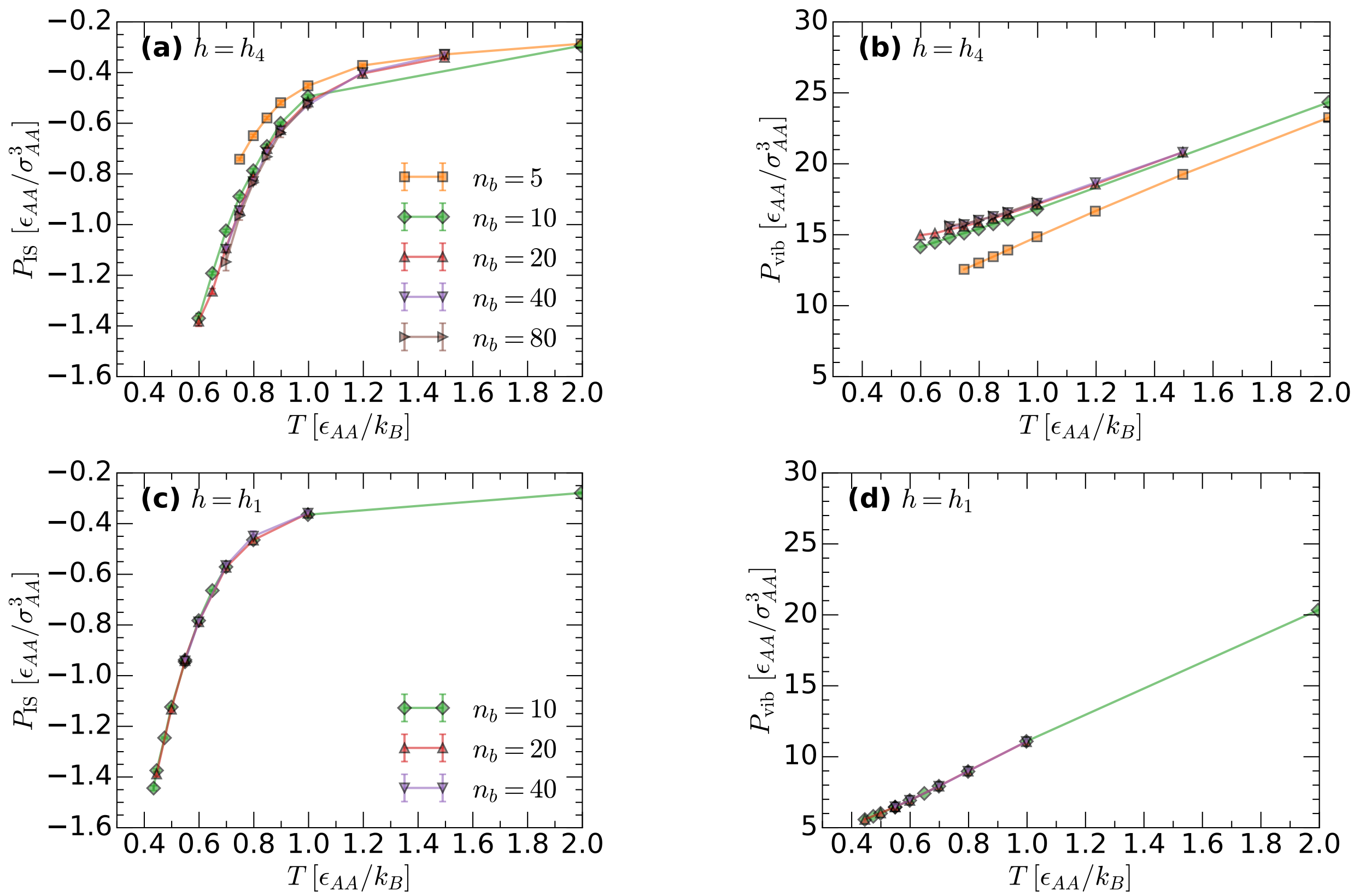

FIG. S5. (a) Inherent structure pressure $P_{IS}(T)$ and (b) vibrational pressure $P_{vib}(T) \equiv P(T) - P_{IS}(T)$ as a function of temperature obtained from PI simulations of the quantum LJBM with Planck's constant $h = h_4 = 1.0000$, and for $n_b = 5, 10, 20, 40, 80$. (c)(d) Same as (a)(b) for the case $h = h_1 = 0.2474$, and for $n_b = 10, 20, 40$. In both cases, $P_{IS}(T)$ and $P_{vib}(T)$ converge for $n_b \geq 10 - 20$.

S.III. VIBRATIONAL HELMHOLTZ FREE ENERGY: TEMPERATURE DEPENDENCE OF THE PEL VARIABLES $\tilde{a}(T)$ AND $\tilde{b}(T)$

For a quantum liquid with a harmonic RP-PEL in the limit $n_b \rightarrow \infty$, the vibrational Helmholtz free energy $F_{vib}^{harm}(T, e_{IS})$ is given by Eq. 22 of the main manuscript (N and V are constant). In the case of the LJBMs studied, Eq. 22 is well-approximated by Eq. 25. The coefficients $\tilde{a}(T)$ and $\tilde{b}(T)$, defined in Eq. 25 of the main manuscript, for the LJBM studied in the main manuscript (with Planck's constant $h = h_4$) are included in Fig. S6(a)(b); both PEL variables converge for $n_b \geq 20$. Similar results hold for the LJBM with $h = h_1$. In this case, $\tilde{a}(T)$ and $\tilde{b}(T)$ converge for $n_b \geq 10$; see Fig. S6(c)(d).

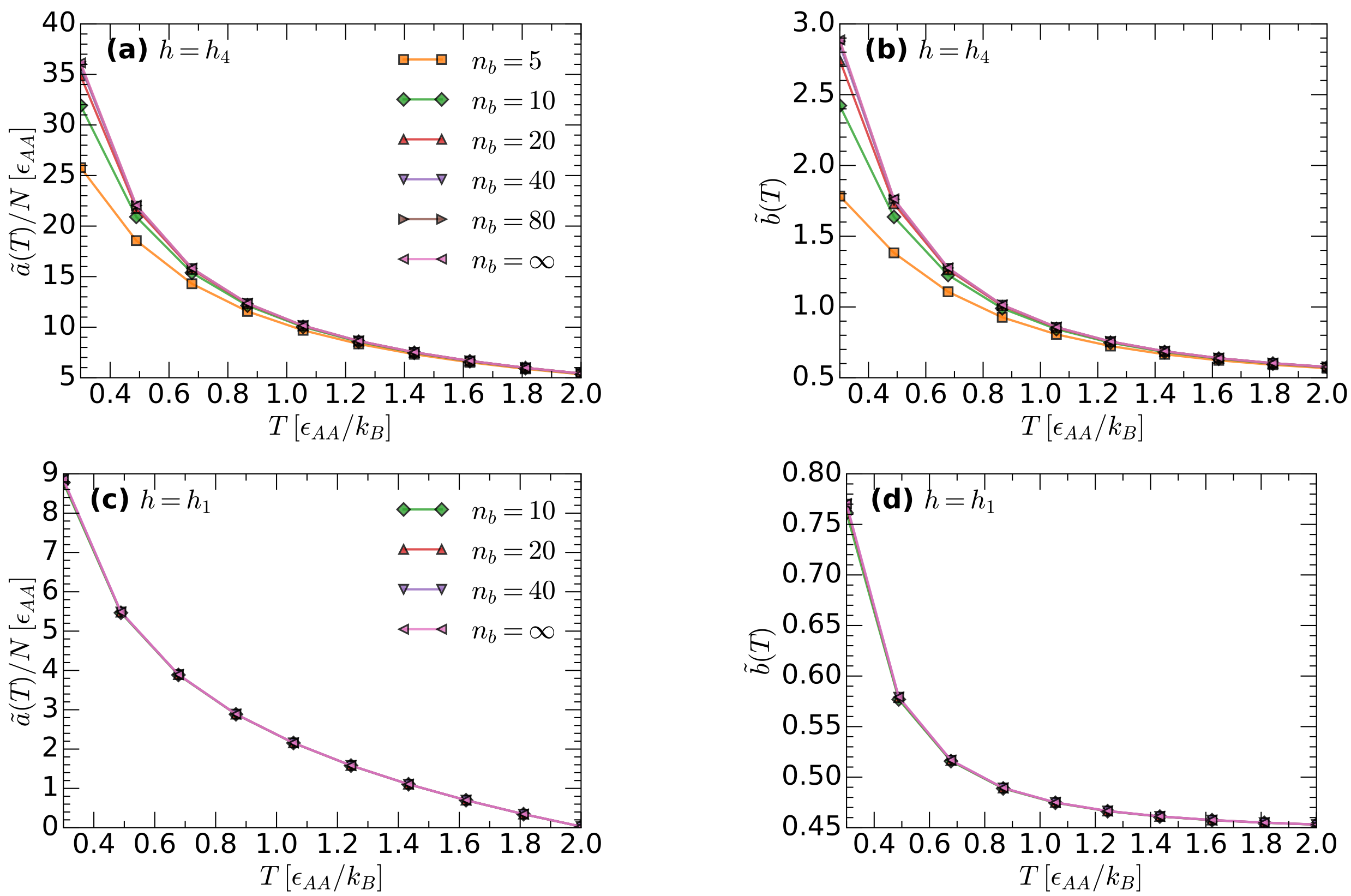

FIG. S6. Temperature dependence of the PEL-variables $\tilde{a}(T)$ and $\tilde{b}(T)$ defined in Eq. 25 of the main manuscript. (a)(b) Results are obtained from PI simulations of the quantum LJBM with Planck's constant $h = h_4 = 1.0000$, and for $n_b = 5, 10, 20, 40, 80$. (c)(d) Same as (a)(b) for the case $h = h_1 = 0.2474$, and for $n_b = 10, 20, 40$. In both cases, $\tilde{a}(T)$ and $\tilde{b}(T)$ converge for $n_b \geq 10 - 20$.

S.IV. TESTING THE HARMONIC APPROXIMATION FOR THE LJBMS WITH $h = h_1$ AND h_4

In this section, we show that the RP-PEL of the weakly quantum LJBM (PI computer simulations with $h = h_1$) is harmonic while it is anharmonic for LJBMs with relevant quantumness (PI computer simulations with $h = h_4$). Figs. S7(a)(b) show the vibrational energy of the LJBMs, $E_{vib}(T)$, obtained from PI computer simulations with $h = h_1,\ h_4$ (symbols; taken from Fig. S3(b) and Fig. 3(b) of the main manuscript). The dashed lines are the corresponding predictions from the PEL formalism for quantum liquids based on the Gaussian and harmonic approximations for a finite value of n_b,

$$E_{vib}(n_b, N, V, T, E_{IS}) = 3Nn_bk_BT + \left(\frac{\partial \mathcal{S}(n_b, N, V, T, E_{IS})}{\partial \beta}\right)_{N,V,E_{IS}} \tag{S1}$$

As shown in Ref. [1], Eq. S1 follows from the expression $E_{vib} \equiv E - E_{IS}$, where $E = \left(\frac{\partial(\beta F)}{\partial \beta}\right)_{N,V}$, and Eq. 15 of the main manuscript. Fig. S7(a) shows that for $h = h_4$, the results from PI simulations (symbols) deviate from the corresponding prediction of the PEL formalism (dashed-lines). This is the case for all the studied value of $n_b = 5,\ 10,\ 20,\ 40,\ 80$. Accordingly, for the values of n_b considered, the RP-PEL of the LJBM with $h = h_4$ is not harmonic. Instead, as shown in Fig. S7(b), the results from PI simulations for the case $h = h_1$ (symbols) fully agree with the corresponding prediction of the PEL formalism (dashed-lines), i.e., the RP-PEL of the LJBM with $h = h_1$ is harmonic for all the studied values of $n_b = 10,\ 20,\ 40$.

For comparison, we also include in Figs. S7(a)(b) the predictions for $E_{vib}(T)$ from the PEL formalism (based on the Gaussian and harmonic approximations) for the case $n_b \to \infty$ (solid magenta lines); specifically,

$$E_{vib}(N, V, T, E_{IS}) = \left\langle \sum_{i=1}^{3N} \left(\frac{\hbar\omega_{i,0}}{2} + \frac{\hbar\omega_{i,0}}{(e^{\beta\hbar\omega_{i,0}} - 1)}\right)\right\rangle_{E_{IS}}. \tag{S2}$$

Eq. S2 can be obtained by following the same procedure in Ref. [1] but using Eq. 22 ($n_b \to \infty$) instead of Eq. 15 (finite n_b). As expected, Fig. S7(a) shows that, for $h = h_4$, the predictions from the PEL formalism for $E_{vib}(T)$ obtained for finite values of n_b (dashed lines), Eq. S1, converge with increasing n_b to the expression given by Eq. S2 ($n_b \to \infty$; magenta line). In the case $h = h_1$, the LJBM is harmonic for all the values of n_b studied. In this case, the predictions for $E_{vib}(T)$ obtained using Eq. S1 (finite n_b, dashed lines) and Eq. S2 ($n_b \to \infty$,

solid magenta line) overlap.

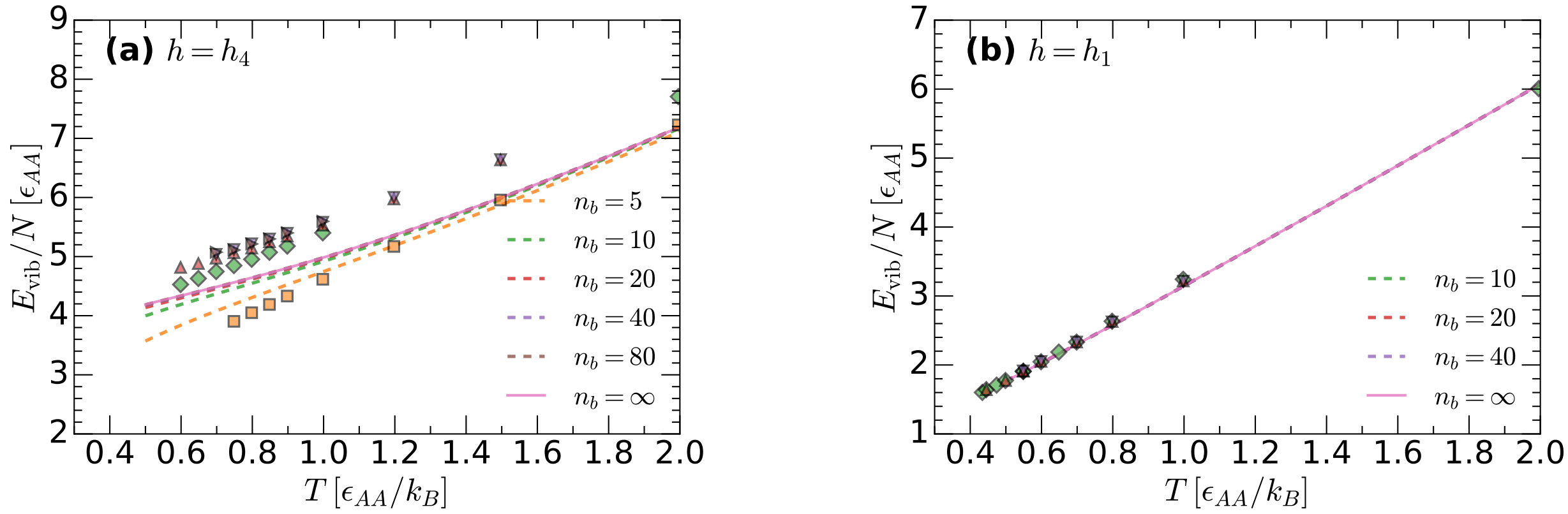

FIG. S7. Vibrational energy $E_{vib}(T)$ as a function of temperature for the LJBMs studied with Planck's constant set to (a) $h = h_4$ and (b) $h = h_1$. Symbols are results from PI computer simulations for different values of beads per ring-polymer, n_b [taken from Fig. S3(b) and Fig. 3(b) of the main manuscript]. The dashed lines are the corresponding predictions for $E_{vib}(T)$ from the PEL formalism for a finite value of n_b, Eq. S1. The solid magenta line is prediction from the PEL formalism for $E_{vib}(T)$ for $n_b \to \infty$, Eq. S2. In (b), all dashed lines overlap with the solid magenta line.

The LJBM with $h = h_1$ is harmonic and hence, $F_{vib} = F_{vib}^{harm}$ (Fig. S4). In contrast, the LJBM with $h = h_4$ is anharmonic, i.e., $F_{vib} = F_{vib}^{harm} + F_{vib}^{anh}$. As shown in the main manuscript, F_{vib} and F_{vib}^{harm} both converge as $n_b \to \infty$ and hence, F_{vib}^{anh} must converge as well. This implies that the anharmonic contributions to vibrational properties also converge in the limit $n_b \to \infty$. As an example, Fig. S8 shows the behavior of $E_{vib}^{anh}(n_b, T)$ as a function of T for all the values of n_b studied.

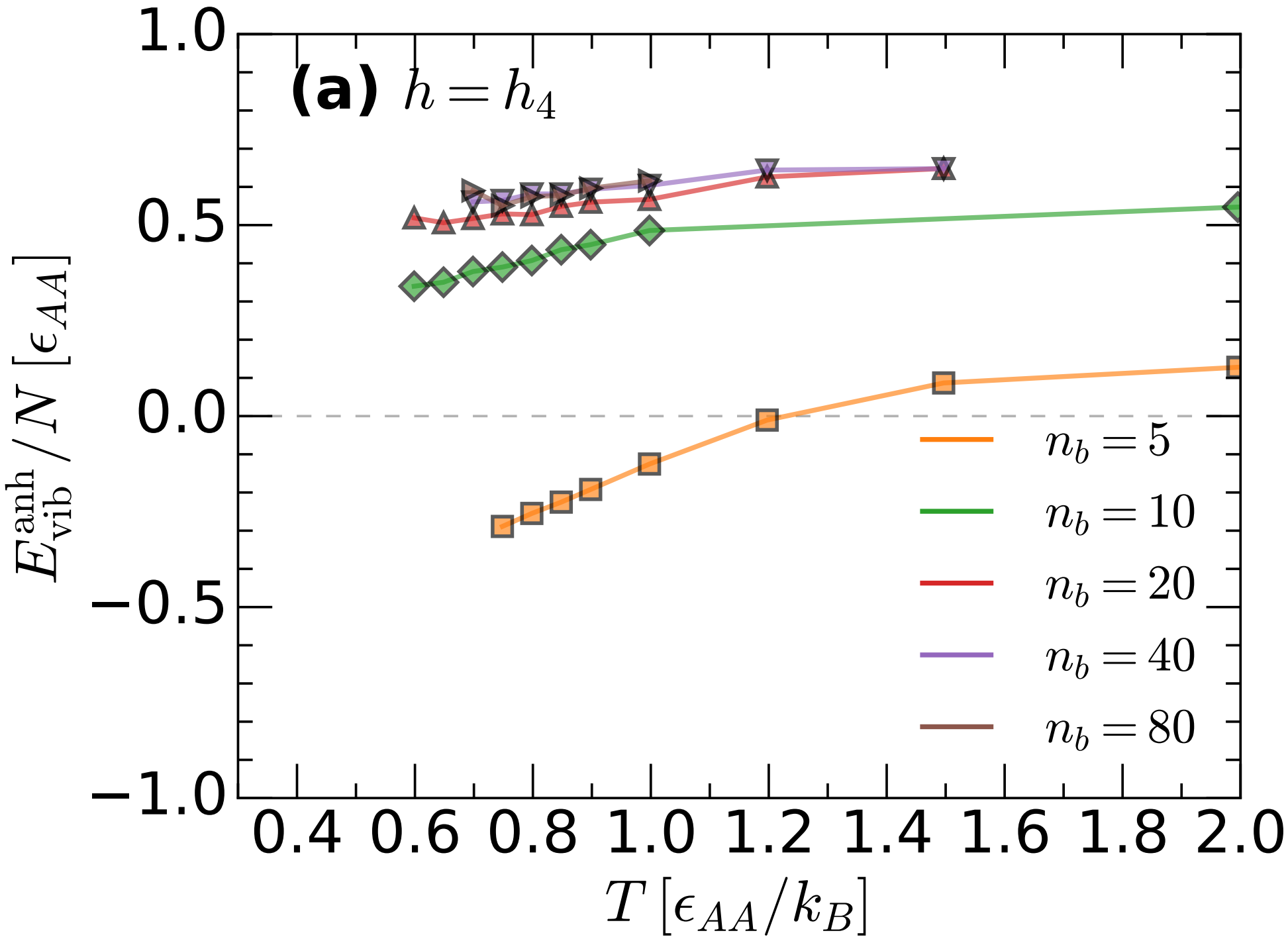

FIG. S8. Anharmonic contribution to the vibrational energy, $E_{vib}^{anh} = E_{vib} - E_{vib}^{harm}$, as a function of temperature and for the studied values of n_b. Results are for the LJBM with Planck's constant $h = h_4$. $E_{vib}^{anh}(T, n_b)$ converges as $n_b \to \infty$.

[1] Y. Zhou, A. Eltareb, G. E. Lopez, and N. Giovambattista, Configurational entropy and Adam-Gibbs relation for quantum liquids, Nature Communications **17**, 4843 (2026).
